# Quantitative dynamics of neural uncertainty in sensory processing and decision-making during discriminative learning

**DOI:** 10.1038/s12276-025-01456-7

**Published:** 2025-05-07

**Authors:** Soonho Shin, Joonsu Oh, Sun Kwang Kim, Yong-Seok Lee, Sang Jeong Kim

**Affiliations:** 1 Department of Physiology, Seoul National University College of Medicine, Seoul, Republic of Korea; 2 Department of Biomedical Sciences, Seoul National University College of Medicine, Seoul, Republic of Korea; 3 Memory Network Medical Research Center, Neuroscience Research Institute, Wide River Institute of Immunology, Seoul National University College of Medicine, Seoul, Republic of Korea; 4 Department of Electrical and Computer Engineering, University of Toronto, Toronto, Ontario Canada; 5 Department of Physiology, College of Korean Medicine, Kyung Hee University, Seoul, Republic of Korea

**Keywords:** Decision, Perception, Cortex

## Abstract

Uncertainty is crucial in sensory processing, necessitating further quantitative research on its neural representation in the sensory cortex. Here, to address this need, we used a deep learning approach to quantify uncertainties in neural activity from the forelimb area of the primary somatosensory cortex (fS1) during a vibration frequency discrimination task, introducing a transformer model designed to decode neural data not consistently tracked over time. Our model shows that the neural representation of fS1 encodes uncertainties not only from vibratory stimuli but also from decision-making processes, emphasizing its crucial role across various biological contexts. We confirmed that uncertainty decreases as learning progresses and increases with interruptions in learning. In line with predictions from previous studies, we also observed that uncertainty is high at psychometric thresholds. Furthermore, high uncertainty correlates with incorrect decisions, and we have identified dynamics in uncertainty between previous and current trials. Such findings underscore the evolving role of fS1 in assessing uncertainty for the brain’s downstream areas as learning progresses.

## Neural uncertainty shapes decision-making in sensory cortex

This study explores how the brain deals with uncertainty, which is crucial for learning and decision-making. Yarin Gal introduced a new method using Monte Carlo dropout (MCD) to better measure uncertainty in neural networks. This study applied MCD to understand how mice process sensory information in their brains, specifically in the primary somatosensory cortex (fS1). Researchers used 15 mice, conducting experiments with two-photon calcium imaging to observe brain activity. The study used a neuron transformer model, a type of deep learning model, to decode neural activity and measure uncertainty. Results showed that, as mice learned, their brain’s uncertainty decreased, but it increased when learning was interrupted. The study concludes that understanding neural uncertainty can improve insights into brain function and has potential implications for studying disorders such as schizophrenia and autism.

This summary was initially drafted using artificial intelligence, then revised and fact-checked by the author.

## Introduction

Uncertainty plays a fundamental role in how we understand and interact with the world^1,2^. Organisms’ sensory inputs inherently produce noisy signals due to sensor noise and intrinsic neuronal noise^3^, leading to variability. The brain assesses this uncertainty on the basis of past experiences, allowing flexible interpretation of inputs^4^ and influencing subsequent actions^5^. It is critical for adapting to changing environments and navigating unpredictable situations. Uncertainty is also deeply intertwined with the brain’s learning mechanisms.

In previous neuroscience studies, primarily using correlational or code-driven methods^4^, have provided indirect and correlative insights^6^. However, these approaches often involve limited situations or restrictive assumptions, such as difficulties in capturing temporal dynamics, inability to handle variable neural populations, and unrealistic assumptions about neural noise, highlighting the need for more general models for effective analysis^7^. While previous investigations in neuroscience have been valuable, they often lack a quantitative approach, relying primarily on qualitative analyses^4^.

In the domain of machine learning, there has been significant advancement regarding insights into uncertainty. Yarin Gal proposed a new method that addresses the high computational demands and inherent limitations of traditional Bayesian neural networks^8,9^. This is achieved by applying the Monte Carlo dropout (MCD) technique not just in training but also during the inference phase, thus enabling a more practical and robust quantification of uncertainty through the measurement of variance in inference outcomes^10^.

Research on uncertainty has focused on various brain areas, including the amygdala^11^, orbitofrontal cortex^11,12^, prefrontal cortex^13,14^, visual cortex^15^, somatosensory cortex^16^ and others. The primary somatosensory cortex (S1), in particular, has emerged as a prime candidate due to its critical role in processing sensory information and its potential role in learning. The S1 undergoes significant changes during the process of learning, as it not only represents simple low-level stimuli but also encompasses cognitive information necessary for learning, such as reward^17^, behavioral choice^18^ and attention^19^. The vibration frequency discrimination task involves participants distinguishing different vibration frequencies and is particularly relevant for exploring sensory processing and decision-making^20^. These elements are crucial in understanding the role of uncertainty in cognitive processes^4^. As a result, this behavioral task provides the means for a detailed quantitative assessment of uncertainty.

The use of MCD variance to measure neural uncertainty aligns with how the brain’s complex network of neurons processes information stochastically. The dropout process in MCD mimics this stochasticity by randomly deactivating pathways in the neural network model, thus providing a computational analog to the uncertainty observed in biological neural networks. Moreover, the ability of MCD to measure uncertainty and produce different outcomes for the same input can be likened to how organisms behave differently on the basis of their evaluation of uncertainty in response to the same stimulus. For instance, in the visual cortex, current models have shown that uncertainty in the stimulus leads to increased gain variability—a measure of how much a neuron’s response varies with each trial^21^. This mirrors the findings from evaluating the neural representation of uncertain stimuli in fS1 through MCD, where high variability in model results suggests high uncertainty. Therefore, this theoretical background supports the idea that our proposed model can significantly mimic the brain’s process from receiving neural representations in fS1 to making decisions.

Uncertainty not only drives learning but also changes dynamically throughout the process. By quantifying uncertainties in data from the fS1 during the learning process, our study demonstrates the evolving nature of uncertainty in the brain’s decision-making mechanisms. We show that uncertainty adaptively changes as learning progresses, decreasing with ongoing training but increasing when learning is interrupted. In addition, we observed that higher levels of uncertainty are frequently associated with incorrect decisions. In line with predictions from previous studies, we observed significant uncertainty at psychometric thresholds. Furthermore, we identified dynamics in uncertainty across trials, illustrating how previous experiences and uncertainties influence current decision-making. Through this, we investigated the role of uncertainty in sensory processing as it relates to decision-making and learning. Moreover, this research has introduced an innovative methodology for the comprehension of uncertainty within the brain.

## Materials and methods

### Animals

All experimental procedures involving animals were conducted under the approval of the Institutional Animal Care and Use Committee of Seoul National University. In total, 15 mice, aged 8–16 weeks, were used in the experiments. The mice used in the experiments were transgenic, characterized by the expression of GCaMP6f in excitatory neurons, and were identified as the Slc17a7;Ai93;CaMKIIa-tTA lines from The Jackson Laboratory (#024108 and #023527)^22^. In this experiment, we excluded any mice identified with aberrant epileptiform activity in previous research^23^. The animals were housed with their littermates in a controlled environment, with regulated temperature and humidity, under a reverse 12-h light–dark cycle. All behavioral assessments were conducted during the animals’ active (dark) phase.

### Surgery

Surgical procedures for two-photon (2P) calcium imaging were performed on mice aged 6–7 weeks. Anesthesia involved an intraperitoneal injection of zoletil and xylazine (3 mg/kg and 10 mg/kg, respectively), along with dexamethasone (2 mg/kg). In a stereotaxic apparatus with a heating pad and eye protection, the scalp was incised, and a custom titanium headplate with a central ring-shaped aperture was secured to the right fS1 (0.25 mm anterior, 2.25 mm lateral from bregma) using 3 M Vetbond and dental adhesive resin cement (Super-Bond C&B, Sun-Medical). After the cement cured, a craniotomy was performed at the fS1 coordinates with a craniobot^20^, creating a 3.4-mm-diameter cranial window. Three round glass coverslips (one 5 mm and two 3 mm, each 150 μm thick) were layered, bonded with ultraviolet-curable optical adhesive (Norland Optical Adhesive 71) and secured with dental cement. After a week of recovery, behavioral training commenced.

### Behavioral procedures

All behaviors were controlled through custom-written LabVIEW code. All behavioral experiments were conducted in the presence of white noise at 75 dB.

#### Handling

A daily 10-min handling routine was implemented for 3 days.

#### Habituation

Over 3 days, mice were habituated to being head-fixed while maintaining proper posture. They were positioned with their right forepaw on a fixed platform and their left forepaw on a vibration platform, separated by a 2-cm gap from a cylindrical restrainer supporting their body. Habituation sessions lasted 10, 20 and 40 min, respectively.

#### Vibration acclimation

After maintaining proper posture during habituation, the mice were acclimated to vibration stimuli to prevent movement upon stimulation. Over 3 days, their left forepaw received 200 randomized vibrations, varying in frequency (200–600 Hz at 40-Hz intervals) and displacement (3 μm). Each stimulus lasted 0.25 s, with intervals ranging randomly from 2.5 to 4.5 s.

#### Pretraining imaging

On the final day of acclimation, 2P imaging captured GCaMP6f calcium activity in response to stimuli ranging from 200 Hz to 600 Hz, without movement. In these areas, 200 Hz and 600 Hz vibrations were administered in 25% of cases each, an unexpected reward (UR) occurred 5% of the time and the remaining 45% involved varied vibrations between 200 Hz and 600 Hz, totaling 200 imaging instances.

#### Water restriction

Water restriction was initiated on the pretraining day. Water was made available only during the behavioral tests on the apparatus; however, should the daily intake fall below 1.2 ml, additional water was provided manually to guarantee a minimum intake of 1.2 ml. On nonexperimental weekends, the mice were kept hydrated by administering 1.5 g/day of Solid Drink (Triple A Trading) per mouse.

#### Lick shaping

Starting the day after initiating water restriction, we began a 3-day lick shaping process. Initially, we encouraged licking by holding the lick port, calibrated to release 6–8 μl per lick, to the mouse’s mouth. Gradually, we trained the mice to lick from a distance by moving the port further away.

#### Vibration frequency discrimination task

The vibration frequency discrimination task lasted 8 days, with each day consisting of 200 trials during 2P imaging sessions, complemented by additional sessions to ensure adequate water intake. The task was controlled via LabVIEW, capturing all data. Each trial included a prestimulus phase (1 s), stimulus delivery (0.25 s), a delay phase (0.25 s), a response window (1.5 s) and a poststimulus phase (1 s), with intertrial intervals ranging from 0.5 to 2.5 s. During each session, 2P imaging and video capture spanned from the prestimulus to the poststimulus phase. The ‘go’ stimulus was a 600-Hz, 3-μm vibration, the ‘no-go’ was 200 Hz, 3 μm, and a ‘probe’ stimulus varied in frequency by 40-Hz intervals at 3 μm. Random events included unpredicted rewards (water without stimulus) and reward omissions (ROs; no reward after a go). The sound of the solenoid valve signified unpredicted rewards, prompting licks. Successful go responses resulted in a ‘hit’ and a 6–8 μl water reward. After 200 trials, an additional session ensured either a minimum of 1.5 ml water intake or ended after five consecutive misses. If intake was below 1.2 ml, water was manually provided. Stimulus distribution in imaging sessions was 36% go, 36% no-go, 25% probe, 1% RO and 2% UR. Probe frequencies ranged from 240 Hz to 560 Hz, distributed as 25%, 26%, 25% and 4% for the rest. In additional sessions, stimuli ratios were 48% go and no-go and 4% probe, with no RO or UR events. Probes were evenly presented at about 11% across frequencies.

### Behavioral apparatus

The behavioral experiment setup was configured as follows. We designed and three-dimensionally (3D) printed the setup, including the fixed platform and cylindrical restrainer. In addition, we custom-designed an aluminum structure for head fixation. The lickometer (Noldus) was linked to the lick port (JD-S-124, oral zonde needle, Jeungdo Bio & Plant), which itself was affixed to an articulated arm through a custom 3D-printed connector, enabling the precise detection of licks. This signal was acquired using a data acquisition card (PCIe6321, National Instruments). Water was supplied through a solenoid valve (LVM11-6A-1, SMC Pneumatics) connected to a 10-ml syringe cylinder using a urethane tube, with the assembly secured by 3D printing, and the solenoid valve and lick port also interconnected with a urethane tube. Lick signals and solenoid valve operations were acquired and controlled using custom-written LabVIEW code.

#### Vibration stimuli

The vibration stimulus was delivered similarly to previous study^24^, utilizing a 3D-printed platform wrapped with sandpaper, which was connected to a piezo actuator (P-841.2, Physik Instrumente). The piezo actuator generates vibrations through a voltage supplied by the LabVIEW and NI Board via an amplifier module (E-501, E504), and its displacement was accurately regulated in a closed-loop manner through a sensor-control module (E-509S1), preventing stimulus alteration caused by the force from the mice. The sinusoidal stimulus was configured to increase linearly over the first 0.025 s and then decrease linearly for the last 0.025 s at the end.

#### Video acquisition

Facial and bodily images were captured at 160 fps under a 940-nm ring light (NV-R-D-940, Nanum Vision) using a GigE mono camera (MG-D030B, Crevis) equipped with a 940-nm filter (BN940-25.4, MidOpt) and lens (C-mount lenses, Computar). The acquisition was conducted via a custom-written LabVIEW code.

### Behavioral analysis

#### Performance analysis

In the discrimination task, from day 1 to 8, we calculated the performance metric using

$${\rm{Perfomance}}\;{\rm{metric}}=\frac{{\rm{Hit}}{\#}+{\rm{CR}}{\#}}{{\rm{Hit}}{\#}+{\rm{Miss}}{\#}+{\rm{CR}}{\#}+{\rm{FA}}{\#}},$$

which encompasses data from both the imaging sessions and additional sessions.

#### Psychometric curve

Responses to vibration stimuli ranging from 200 to 600 Hz were collected daily and analyzed for the first 3 days (naive) and last 3 days (expert), using a generalized linear model fit separately for imaging and additional sessions. The generalized linear model, executed with the MixedPsy package in R (ref. ^25^), extends linear regression for nonnormally distributed responses. ‘Lick’ and ‘no lick’ responses, assumed binomial, were analyzed with vibration frequency as the explanatory variable using a probit link function to assess its impact on outcomes.

#### Response time

Response time was analyzed as the duration from the presentation of the stimulus to the moment of licking, examining the times for hits, probe licks and false alarms (FAs).

#### Premovement trials

Using DeepLabCut^26^, we trained the model to identify the position of the left paw in mouse face videos, subsequently acquiring the coordinates. Trials were classified as ‘premovement’ if the coordinates showed a movement exceeding 60 pixels (approximately 3 mm) within a 0.5- to 1-s window during the prestimulus phase. These trials were excluded from the following deep learning analysis.

### 2P calcium imaging

For in vivo 2P calcium imaging, we used a 2P microscope (FVMPE-RS, Olympus) equipped with a water immersion objective lens (XLPLN25XWMP2, Olympus). The Ti:sapphire laser (Chameleon, Coherent) was set to 900 nm to excite GCaMP6f. We imaged pyramidal cells in layer 2/3 of the fS1 and recorded views responsive to vibration during the PreTraining session, striving to capture the same view on subsequent training days. Images (512 × 500 pixels, 0.99 μm per pixel) were acquired using FLUOVIEW (Olympus) at 30 fps via a trigger controlled by custom-written LabVIEW code.

### Calcium data analysis

We acquired the temporal traces of fluorescence activity driven by calcium using CaImAn^27^. The activity traces from each neuron during the prephase, or the baseline, were utilized to deduce the *z* scores spanning from the stimulus phase to the response window, analyzing them per trial, stimulus or response. Furthermore, to compare the neural activity induced by licking with that resulting from vibration stimuli, we realigned the activity traces with the times of licking set to zero.

Go neurons were classified on the basis of the following criteria: (1) presence of activity with a *z* score above 2 within +200 ms (6 frames) in more than 60% of Go trials; (2) inclusion in the top 5% of the area under the curve in the receiver operating characteristic curve from permutation tests; and (3) no activity in FA and UR trials. A neuron was defined as a Go neuron if it met all three conditions. Lick neurons were categorized on the basis of these criteria: (1) presence of activity with a *z* score above 2 within +200 ms (6 frames) in more than 60% of licking trials; and (2) inclusion in the top 5% of the area under curve in the receiver operating characteristic curve from permutation tests. A neuron was defined as a Lick neuron if it met both conditions.

### Neuron transformer

Our neuron transformer modifies the widely used transformer architecture^28^ to process variable-length sequence data, implemented in PyTorch without positional encoding to learn neural population patterns. Existing methods for measuring neural uncertainty, such as trial-by-trial variability and the Fano factor, struggle with populational encoding analysis. Furthermore, traditional machine learning methods and Bayesian models require consistent input dimensions, which makes it challenging to handle variable neural populations and complicates the analysis of learning courses. To address these challenges, we have adopted a transformer model. In comparison with Bayesian neural networks, the method developed by Yarin Gal is more cost-effective and is supported by robust mathematical validation, which influenced our decision to use this approach^10^. Despite studies^29^ suggesting that models might retain positional information implicitly, our simpler design focuses on pattern learning due to less precise positional data. We trained the model excluding data from specific dates (day 7) or mice, preventing it from learning that date’s positional details and allowing assessment of its decoding accuracy. This confirmed our model’s capability to decode effectively without positional information, as it learned patterns sufficiently (Fig. 2i). We conducted a comparison of our neuron transformer with traditional methods in the Supplementary Information and found that our model not only performs better but also more accurately fits the biological context.

#### Datasets

Utilizing CaImAn for preprocessing 2P images, we incorporate preprocessing steps including neutrophil subtraction and movement correction to derive the neural activities. Subsequently, these activities are min–max scaled and then segmented into nine-frame portions, specifically capturing frames 29–37 from each trial. Each segment is labeled with trial statuses (for example, stimulus and response), forming the structured datasets. For stimulus, labels were assigned with 200 Hz as 0 and 600 Hz as 1, whereas, for response, ‘no lick’ was labeled 0 and ‘lick’ was labeled 1. The proportions for the train, validation and test sets are 76%, 19% and 5%, respectively.

#### Architecture

The model is composed of a sequence of processing blocks, beginning with a signal encoder and culminating in the final output layer (Fig. 2a).

##### Signal encoder (feed forward)

The architecture starts with a signal encoder, a simple feed-forward network that uses data into a desired latent dimension size (9–30).

##### Transformer block

Our architecture features two identical transformer blocks connected in sequence, each processing neural activity for one trial per mouse. Key components include layer normalization, Q, K, V projections for self-attention, dropout (used here to quantify uncertainty), standard feed-forward networks, GeLU activation and Add & LayerNorm for residual connections.

##### Post-transformer layers

Postprocessing includes max pooling to condense transformer output to a manageable size and a subsequent feed-forward network for further processing.

##### Training

These data were subjected to a training regimen with a batch size of 64 for training, 16 for validation and 16 for testing. We established the dropout probabilities for embedding, residual and attention at 0.1. Furthermore, we instituted a learning rate of 0.0001 over 20,000 iterations, preserving the model exhibiting the minimal cross-entropy loss during validation for ensuing testing procedures. The training was conducted using the Adam optimizer across all architectures mentioned above.

##### Accuracy statistical analysis

Decoding accuracy underwent analysis of variance (ANOVA) testing; significant results (*P* < 0.05) led to further analysis with Tukey’s honestly significant difference (HSD) test to assess significance between pairs.

### Uncertainty quantification

In our neuron transformer model, we achieved uncertainty quantification (UQ) by applying dropout consistently across training and testing phases, performing multiple inferences with dropout enabled on the same data instance.^10^. Subsequently, we calculated variance values from these inferences to use as measures of uncertainty.

We assessed uncertainty using *k*-fold cross-validation, splitting the dataset into 20 folds, each isolating trials with 200 Hz or no lick (label 0) and 600 Hz or lick (label 1), excluding premovement and UR instances. In each fold, 80% of data were used for training and 20% for validation. Therefore, the proportions for the train, validation and test sets are 76%, 19% and 5%, respectively. This training setup enabled UQ by inferring values 1000 times per test dataset, repeating for all 20 datasets and across 10 different runs using varied random seeds for robust analysis.

### Uncertainty statistical analysis

In our analysis for Figs. 2–5, we first conducted ANOVA tests. Where *P* values were less than 0.05, Tukey’s HSD test was applied to each pairwise comparison. In addition, when assessing differences of a particular dataset from the rest, we initially performed Bonferroni correction followed by independent two-sample *t*-tests. The significance level was set at 0.05 for all tests.

### Correlation analysis between *d*′ and uncertainty

We compiled a dataset containing behavioral trial outcomes and uncertainty metrics from mice performing a go/no-go task. Data were grouped by mouse and experimental day, with each group segmented into 50-trial blocks to facilitate analysis of behavioral performance and uncertainty stability. *d*-prime (*d*′), a measure from signal detection theory for distinguishing signals from noise, was calculated using *d*′ = *Z*(hit rate) − *Z*(false alarm rate), where hit rate and false alarm rate include a 0.5 correction for zero occurrences. We assessed the relationship between *d*′ and uncertainty using Pearson’s correlation coefficient to understand the linear association between these variables.

## Results

### Adequate learning achievement in mice during the vibration frequency discrimination task

**Fig. 1:**
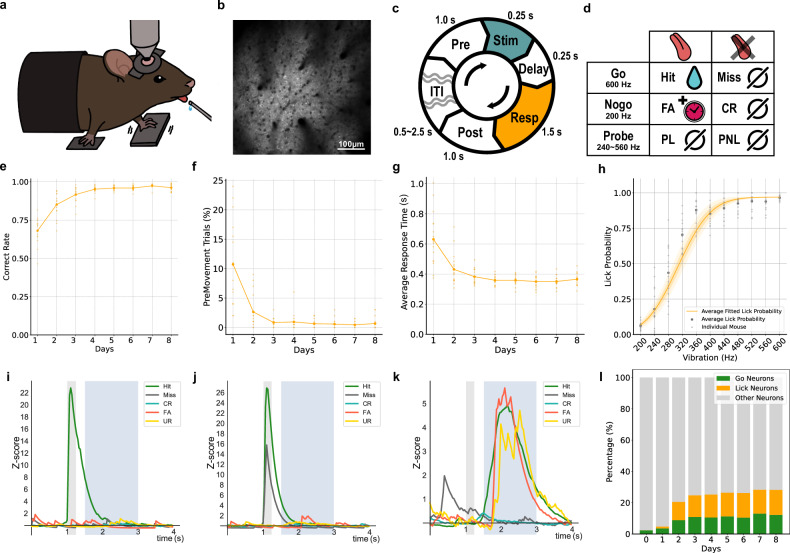
Functional dynamics of layer 2/3 pyramidal cells in fS1 during the vibration frequency discrimination task. **a** The behavioral experiment setup. **b** A time-averaged image from 2P calcium imaging of fS1 layer 2/3. **c** The layout of individual trials. These trials form a daily regimen of 200 imaging sessions and additional sessions. **d** Contingent outcomes based on responses to each stimulus. FA false alarm; CR correct reject; PL probe lick; PNL probe no lick. **e** An increase in the correct rate in mice behavior as learning progresses (each point from e to g represents an individual mouse). **f** The proportion of trials with left forelimb motion just before or during stimulus delivery. **g** A response time reduction in the initial 3 days of learning, followed by a consistent level. **h** Psychometric curve. **i** An example of an averaged calcium trace from a single neuron responding only to hits (gray shaded: stimulus phase; blue shaded: response window). **j** An example of an averaged calcium trace of a neuron responding to 600-Hz stimuli. **k** An example of an averaged calcium trace of a neuron activated by licking. **l** Changes in the proportion of go and lick neurons over days.

We trained head-fixed mice, expressing GCaMP6f in their pyramidal cells, for 8 days to differentiate vibrational stimuli on their left paw according to frequency (Fig. 1a). Each day’s experiment consisted of an imaging session with 200 trials to obtain 2P calcium images (Fig. 1b), followed by an additional session to replenish the deficient water levels. Each trial included a prephase for baseline imaging (1 s), a stimulus phase (0.25 s), a short delay phase (0.25 s), followed by a response window (1.5 s) where the mouse’s response leads to either rewards or punishments, and an additional postphase for further imaging (1 s). An intertrial interval of 0.5–2.5 s is provided between trials (Fig. 1c).

For a 600-Hz, 3-μm stimulus, a go condition was set, prompting mice to lick for a reward (‘hit’), with nonresponders (‘miss’) facing a brief random interval before another attempt (Fig. 1d). Conversely, a 200-Hz, 3-μm stimulus created a no-go condition; not licking resulted in a similar interval (‘correct reject’), while licking led to a 12-s ‘timeout’ period (false alarm, FA). In addition, the task intermittently included probe trials (probe lick and probe no lick), reward omissions (ROs) and unexpected rewards (URs). Probe frequencies varied between 200 Hz and 600 Hz in 40-Hz increments. ROs presented a go stimulus without a reward after licking, and URs involved water delivery without a vibrational cue, identified by the sound of the valve opening.

After 8 days of training, mice reached a 97.4% correct response rate (Fig. 1e). Early in the training, there was a notable decrease in premovement—the movement of the left forepaw just before stimulus onset—ensuring stimuli detection (Fig. 1f). Also, a reduction in response time after the stimulus was recorded (Fig. 1g).

By analyzing responses to go, no-go and probe conditions, we generated a psychometric curve (Fig. 1h). This curve indicates that mice discern the proximity of a probe stimulus to instructed stimuli and respond accordingly. This observation suggests difficulties in decision-making regarding licking when subjected to vibrational stimuli ranging from 240 Hz to 360 Hz, particularly between 280 Hz and 320 Hz, where decisions appear nearly random, indicating high uncertainty. Considering the encoding of choices (responses) in S1 (ref. ^17^), we anticipated a high degree of uncertainty in the encoding of choices in mice’s fS1 when stimuli between 280 Hz and 320 Hz are presented.

### Functional dynamics of layer 2/3 pyramidal cells in fS1 during task

In our analysis, we observed distinct fluorescence patterns in neurons. Neurons responding to the go (600 Hz) stimulus showed different activities; some exhibited reward-expecting activity by responding only in hit scenarios and not in miss cases (Fig. 1i), while others responded to both hit and miss scenarios (Fig. 1j). As learning progressed and miss events decreased, distinguishing between these neuron types became challenging, leading us to collectively classify them as ‘go neurons’. Lick-induced neurons, active during FAs and URs, also showed post-lick activity increases (Fig. 1k). Notably, unlike previous studies^20^ using virus-expressed GCaMP, our transgenic mice showed no responsiveness to no-go (200 Hz) stimuli. Throughout the learning period, the proportion of both go and lick neurons among all observed neurons increased significantly from 3.6% to 12.2% and 1.1% to 15.9%, respectively, with statistical significance (paired *t*-test, *P* < 0.001 from the first to the last day; Fig. 1l).

### Stimulus and response decoding in fS1 using the neuron transformer

Due to the limitations of previous methods discussed earlier, we decided to use a neural network implementing Yarin Gal’s method^10^, as it has been mathematically proven to yield results comparable to those of Bayesian neural networks while being lighter and more practical. Given the inconsistency in the number of neural cells observed from mouse to mouse and from date to date, we ruled out feed-forward neural networks, which need uniform input sizes, and decided to proceed with transformers.

**Fig. 2:**
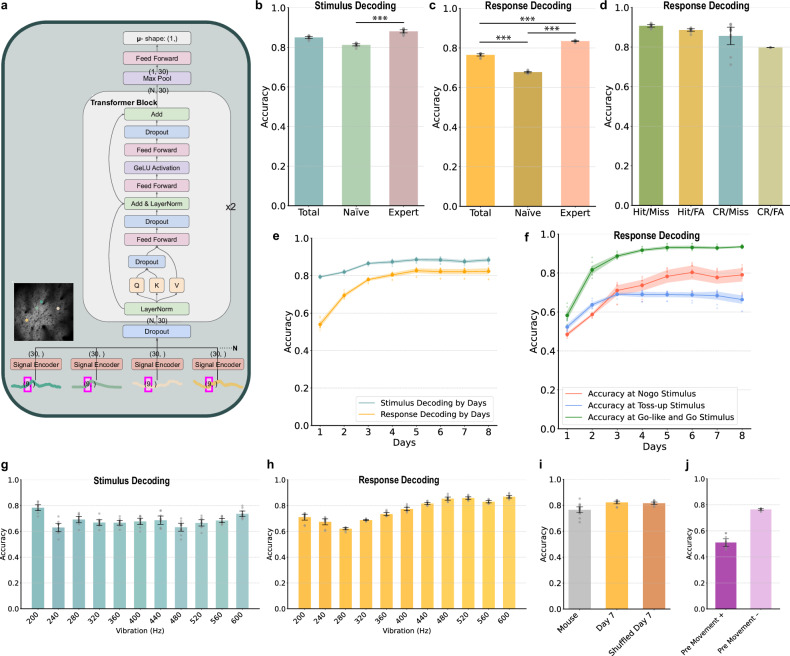
Decoding results of the neuron transformer. **a** The structure of the neuron transformer for UQ. **b** Model accuracy according to stimulus over the ‘total’ 8 days, with ‘naive’ indicating the first 3 days and ‘expert’ the last 3 days. (total 0.850, naive 0.813, expert 0.880) **c** Model accuracy according to response type, lick versus no lick (total 0.765, naive 0.678, expert 0.834) For decoding, the output classes involved are identifying 200-Hz versus 600-Hz stimuli. The chance performance, given two output classes, is 1/2. **d** Decoding results of the model for each response type pair (hit/miss 0.907, hit/FA 0.886, CR/miss 0.856, CR/FA 0.798) The chance performance is 1/2. **e** The decoding performance of stimulus and response across days of training. The chance performance is 1/2. **f** Model accuracy across days for no-go/toss-up (240–320 Hz), go-like (440–560 Hz) and go stimuli. There was a sustained low decoding performance by the model for responses at toss-up frequencies over time. **g** Stimulus decoding according to stimulus frequency. **h** Response decoding according to stimulus frequency. There was a decreased efficacy in decoding responses to vibration stimuli around the psychometric threshold. **i** Excluding one mouse for the test set and using data from all others for training and validation, and running the deep learning model ten times with day 7 of learning as the test set and other days for training and validation. **j** Trials lacking premovement demonstrated satisfactory model performance, contrasting with the insufficient outcomes in trials featuring premovement. The error bars represent the s.e.m.

We developed a neuron transformer (Fig. 2a), an adaptation of the transformer model^27^, to investigate whether stimulus and response can be decoded from the neural population activity in fS1. The model is composed of a signal encoder, two transformer blocks and an output layer. Detailed information about the model is provided in the Supplementary Information.

To exclude fS1 activity linked to licking responses, we analyzed imaging data captured from each trial during stimulus presentation and before the posttraining established minimum response time of 0.273 s, set after day 4. These data covered 9 frames (frames 29 to 37), totaling 0.233 s poststimulus. Trial labels were based on their respective stimulus or response. We processed the neural activities of all neurons for each trial using a signal encoder and then fed them into a transformer block, which adapts to the varying number of neurons.

Using the neuron transformer, we observed that neural population activity during vibration allowed both the stimulus (200 Hz versus 600 Hz) and the response (lick versus no lick) to be decoded. There was an increase in the ability to decode stimuli in the expert period (final 3 days) over the naive period (initial 3 days), confirming the occurrence of learning-based improvement in stimulus differentiation within fS1 (Fig. 2b, *F* statistic 28.35, *P* = 2.33 × 10^−7^, naive–expert: *P* = 3.49 × 10^−6^). Similarly, through response decoding, we verified that responses were represented in fS1 and that this representation became more distinct with ongoing learning (Fig. 2c, *F* statistic 1,373.46, *P* = 6.9 × 10^−28^, naive–expert: *P* = 2.8 × 10^−23^). To address data imbalances among each response type, upsampling was performed by simply duplicating miss trials, allowing the model to effectively distinguish between hit/miss, hit/FA, CR/miss and CR/FA, thus demonstrating precise discrimination by response type. In particular, the analysis through hit/miss and CR/FA revealed accurate choice discernment, and through Hit/FA and CR/miss it confirmed effective stimulus discrimination (Fig. 2d). Through decoding based on response type, it was determined that the model’s improvement over time was not merely due to predicting responses directly from the stimuli. Particularly in distinguishing between hit/miss and CR/FA, the ability to decode different responses for the same stimulus showed that this improvement was not driven by a simple stimulus–response confounding. As previously mentioned, response decoding was feasible using only calcium data collected during the stimulus presentation, excluding neural activity during the response, thereby effectively addressing the potential confounding between stimulus and response.

Upon examining the model’s daily test accuracy (Fig. 2e), we found that stimulus decoding consistently showed high accuracy from the beginning. As mice underwent learning, a slight but significant increase in accuracy was observed (increase from 79.4% to 88.4%, *P* = 7.6 × 10^−14^). This suggests that mice incrementally enhance their encoding for the stimulus as learning progresses. By contrast, the accuracy of response decoding exhibited a rapid ascent within the initial 3 days, culminating in a high degree of precision (increase from 53.9% to 82.3%, *P* = 7.6 × 10^−16^).

We also performed stimulus and response decoding for individual vibration frequencies. For stimulus decoding, we determined the decoding accuracy of a specific stimulus frequency by distinguishing a particular stimulus from the rest using fS1 activity (Fig. 2g; *P*-value matrix: Supplementary Fig. 1a). The results for 200 Hz and 600 Hz showed enhanced decoding performance in comparison with the probe (*P* = 2.6 × 10^−10^ and 0.002 respectively). Meanwhile, in response decoding, the frequencies 240 Hz, 280 Hz and 320 Hz yielded significantly diminished accuracies (Fig. 2h, *P* = 0.0001, 1.2 × 10^−7^ and 0.0004 respectively; *P*-value matrix: Supplementary Fig. 1b). Given these observations, we termed these three probe frequencies toss-up frequencies and undertook a more detailed analysis of them. When examining the response decoding performance for 200 Hz, ~240–320 Hz and ~440–600 Hz (go-like and go frequency) based on the day, all showed an increase over the first 3 days. However, for the toss-up frequency, there was no observed increase after the initial 3 days (Fig. 2f).

We omitted the positional encoder typically used in traditional transformers. This decision was made because calcium imaging cannot capture the exact same plane daily, the presence of identical neurons in the imaging plane is not guaranteed and the number of observable neurons varies each day. Consequently, our approach prioritizes learning from the patterns in neural population activity. We omitted day 7 data from training to use it for testing, demonstrating that our transformer model can decode effectively without positional encoding, by learning patterns efficiently (Fig. 2i). We also evaluated the decoding performance in the model excluding day 7, with the neuron order in the input data shuffled (shuffled day 7). We found no significant difference in accuracy (*t*-test, *P* = 0.537). Similarly, we observed that a model trained by excluding data from a specific mouse also yielded sufficient decoding performance (Fig. 2j). Thus, we confirmed that the model not only accurately predicts both the stimulus and response of the mice but also effectively generalizes neural activity through training.

### Quantification of neural uncertainty in fS1 based on response across the entire learning period

**Fig. 3:**
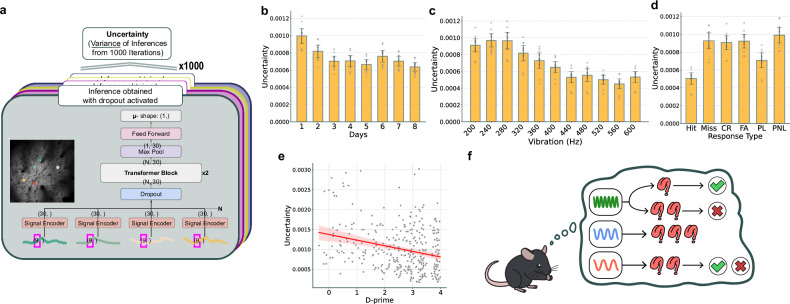
Uncertainty analysis for ‘total’ in choice decoding. **a** The extraction of 9 frames from a total of 120 frames for each neuron’s neural activity in each trial from 2P data. All neurons’ neural activities for each trial were processed through a signal encoder, followed by input into a transformer block, which accommodates varying numbers of neurons owing to its architecture. During the inference process, dropout was enabled for 1,000 iterations to calculate variance and derive uncertainty. **b**–**d** Uncertainties in response decoding over the course of learning (**b**), influenced by stimulus frequency (**c**) and modulated by response type (**d**). Uncertainty decreases with learning (**b**) and shows an increase in incorrect trials (**d**). **e** A significant negative correlation between *d*′ and neural uncertainty, indicating that improved task performance (higher *d*′) is associated with reduced uncertainty. The red line represents the regression line, and the shaded area around it indicates the 95% confidence interval. **f** A conceptual illustration of response uncertainty. There was a correlation of lower uncertainty with correct responses and higher uncertainty with incorrect responses during the go stimulus, and elevated uncertainty at toss-up frequency. In the illustration, the uncertainty related to the response (licking) is depicted for different stimuli: go (green), no-go (red) and probe (blue). The degree of uncertainty is conveyed through the number of tongue-shaped question marks. A check mark indicates a correct result, while an X mark indicates an incorrect result. The error bars represent the s.e.m.

As established, the neural population in the primary sensory cortex is known to encode responses or choices^17,18^. This capability was indicated by our successful decoding of mice’s choices using fS1 data, as shown in Fig. 2c. In our study on the quantification of uncertainty in the fS1 based on response, we modified the transformer model, as described in Yarin Gal’s paper^10^, to compute uncertainty (Fig. 3a). To address this, we integrated a MCD layer into the transformer architecture. This technique allows the model to randomly deactivate some nodes during the prediction process, leading to a variety of outcomes. During the inference process, dropout was enabled for 1000 iterations to calculate variance and derive uncertainty. Therefore, the calculated uncertainty is expressed as a variance value and thus does not have a specific unit, but is rather represented in arbitrary units. We consider that the uncertainty obtained through this method reflects the uncertainty of the neural population, so we will refer to it as neural uncertainty. During the training process of our model, the use of MCD has prevented overfitting, thus enhancing the robustness of the results and enabling the capture of generalized features. This approach effectively addresses potential confounding factors such as neural noise and recording variability.

When presented with vibratory stimuli, the fS1 in mice can determine whether to lick or not to lick. This decision-making process inherently involves a degree of uncertainty. We analyze this under the term ‘response uncertainty’. We measured this neural uncertainty based on ‘lick’ or ‘no lick’ behaviors, using the neuron transformer to assess daily response uncertainty (Fig. 3b; *P*-value matrix: Supplementary Fig. 1c). We noted a decrease in neural uncertainty until day 5 and an increase on day 6, followed by a reduction. The uptick in neural uncertainty on day 6 probably resulted from a pause in behavioral experiments over the weekend. As neither the mice’s behavior nor the model’s accuracy declined on day 6, neural uncertainty does not seem to simply reflect lower performance.

When decoding whether mice licked or did not lick at each vibration frequency to assess response uncertainty, we found higher neural uncertainty at 200 Hz and toss-up frequency. (Fig. 3c; *P*-value matrix: Supplementary Fig. 1d). However, contrary to our prediction that neural uncertainty would be low at 200 Hz and higher near the psychometric threshold, Tukey’s HSD test showed no significant differences when comparing 200 Hz with 240 Hz, 280 Hz and 320 Hz, respectively, yielding *P* values of 0.33, 0.42 and 0.15.

We next examined the neural uncertainty based on the response type. There was a significant reduction of neural uncertainty in both hit and probe lick (Fig. 3d; *P*-value matrix: Supplementary Fig. 1e). Unexpectedly, there was no discernible difference in the neural uncertainty between correct reject and FA.

We also examined the correlation between mouse performance, measured by *d*′, and neural uncertainty (Fig. 3e). The Pearson correlation coefficient between *d*′ and neural uncertainty was found to be −0.273, with a *P* value of 1.36 × 10^−7^. This significant negative correlation shows that better discrimination between go and no-go stimuli corresponds to lower uncertainty, underscoring a clear relationship between task performance and reduced uncertainty.

Through this, the overall neural uncertainty during the learning process can be summarized as illustrated in Fig. 3f. Mice exhibited lower neural uncertainty with the go stimulus, typically resulting in a correct response (hit), and higher neural uncertainty leading to a miss. Surprisingly, for the no-go stimulus, both correct and incorrect responses showed high neural uncertainty. Notably, higher neural uncertainty was observed at the toss-up frequency in probe trials. As neural uncertainty arises from decoding the fS1 neural representation, it seems that the fS1 is involved in processing and representing information in a way that distinctly influences neural uncertainty levels in the subsequent behavioral process.

### The emergence of a difference in neural uncertainty related to choice following the learning process

**Fig. 4:**
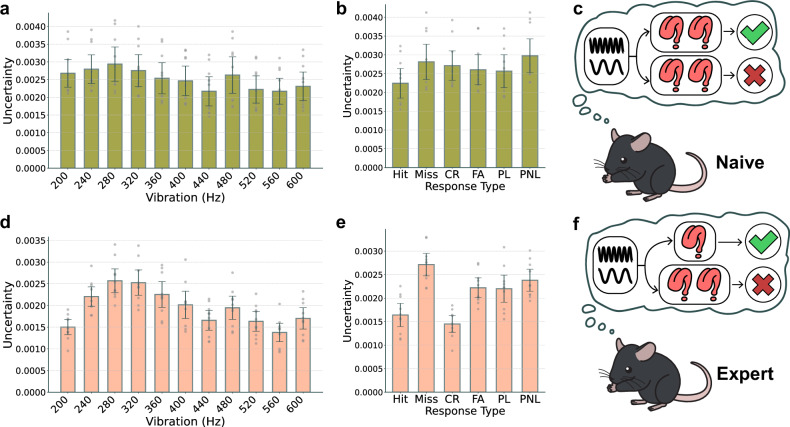
Emergence of response uncertainty variance in expert mice. **a** Frequency-dependent response uncertainty in naive mice with no significant differences observed (ANOVA; *F* statistic 1.523, *P* = 0.142). **b** Type-dependent response uncertainty in naive mice, also lacking statistical significance (ANOVA: *F* statistic 1.317, *P* = 0.270). **c** A concept illustration of uncertainty in naive mice. There were no differences in uncertainty based on vibration stimulus and response type. **d** The variation in response uncertainty in expert mice by vibration frequency, with increased uncertainty at toss-up frequencies and lower uncertainty at 200 and 600 Hz (ANOVA: *F* statistic 9.832, *P* = 3.03 × 10^−11^). **e** Reduced response uncertainty in expert mice during correct trials (hit, CR) (ANOVA: *F* statistic 17.9, *P* = 3.50 × 10^−18^). **f** A concept illustration of uncertainty in expert mice. There were differences in uncertainty according to vibration stimulus and response type. The error bars represent the s.e.m.

Faced with these unanticipated results and the complexities they introduced, we sought to investigate more deeply the changes in neural uncertainty over the course of learning. We hypothesized that the internal state of mice might differ between the initial stages (first 3 days; labeled as naive) of learning and the later stages (last 3 days; labeled as expert). We trained the neuron transformer with data from the first 3 days, labeling the response, to conduct UQ. Unlike in the total phase, in the naive mice’s fS1, there was no significant difference in neural uncertainty based on frequency or response type (Fig. 4a, b; *F* statistic 1.52 for frequency and 1.32 for response type; *P* value of 0.14 for frequency and 0.27 for response type). The neural uncertainty associated with licking behavior, based on stimulus frequency, showed a smaller difference between lick and no lick for the go-like stimulus compared with the total phase (Supplementary Fig. 6). Through this, we were able to verify that, in the brain before proper learning takes place, there is no difference in neural uncertainty related to choice (Fig. 4c).

After sufficient learning had taken place (in expert mice), we conducted UQ on the activity of the fS1 neurons. Upon examining the neural uncertainty for each vibration frequency after thorough learning, the neural uncertainty at 200 Hz significantly diminished (Fig. 4d; *P*-value matrix: Supplementary Fig. 1f), suggesting a more confident response to the no-go stimulus. There was elevated neural uncertainty at the toss-up frequency, which subsequently decreased in the go-like and go stimuli scenarios. This trend mirrors our initial ideas drawn from the psychometric curve.

In evaluating uncertainties based on response types, neural uncertainty manifested prominently lower in hits and correct rejects, showing outcomes that are in sync with intuitive anticipations (Fig. 4e; *P*-value matrix: Supplementary Fig. 1g), diverging from those in the total phase. Interestingly, the neural uncertainty is exceptionally high during misses. Given mice’s innate strong desire to drink water, it is reasonable that the highest neural uncertainty occurred when they did not lick in response to a go stimulus, compared with other types of response. As the mice progressed in their learning to become experts, the overall neural uncertainty decreased. It was observed that lower levels of neural uncertainty led to correct responses, while higher levels of neural uncertainty tended to result in incorrect responses (Fig. 4f). Notably, the neural uncertainty at the toss-up frequency was significantly higher compared with other vibratory stimulus frequencies. This aligns with our intuition and confirms our expectations.

### The dynamics of neural uncertainty based on the relationship between previous and current trials

**Fig. 5:**
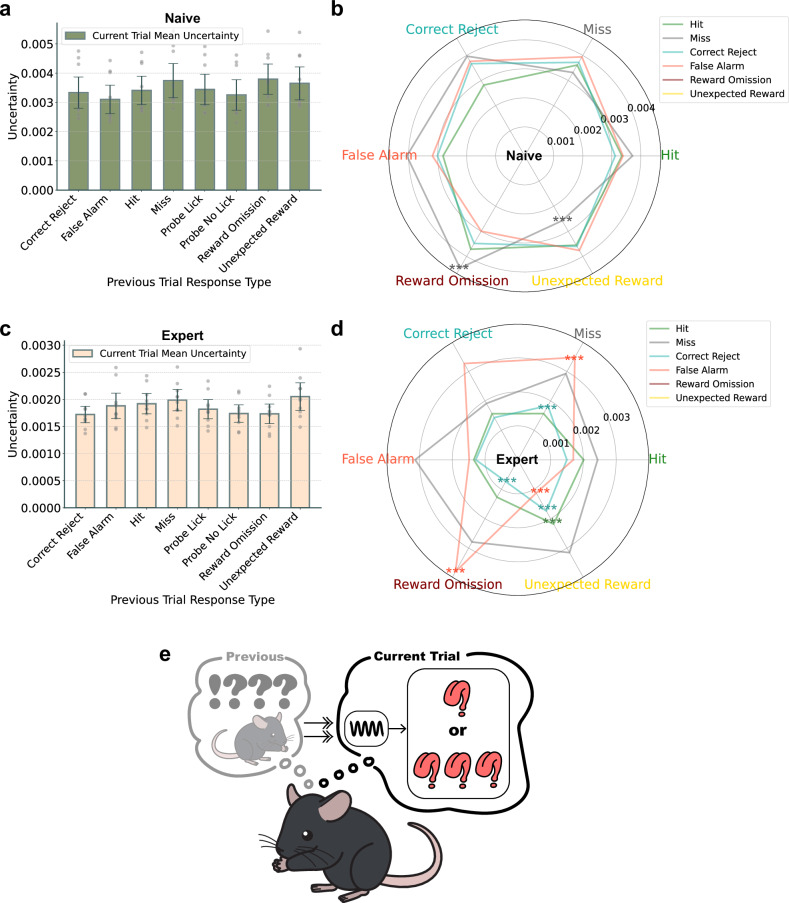
The impact of the intertrial relationship on uncertainty. **a**, **c** Uncertainty across naive (**a**) and expert (**c**) groups in relation to the previous trial’s response type on the current trial’s uncertainty, with no significant statistical differences observed. Subsequent analysis evaluates uncertainty differences for each pair of previous and current trial response types. **b** A spider plot representing uncertainty based on pairs of previous and current trial response types, with axes indicating previous trials and lines differentiated by color to represent current trials. Naive phase uncertainty associated with pairs of response types is presented, with the dynamics between consecutive trial responses detailed. **d** Expert phase uncertainty in response type pairs, highlighting significant variations in uncertainty after unexpected trial sequences. **e** Visualization of uncertainty dynamics influenced by prior trial outcomes. There were changes in mice’s uncertainty in the current trial after unexpected outcomes in the previous trial. The error bars represent the s.e.m.

It is known that neural uncertainty is influenced by prior experiences^30^. Previous studies^17,31–33^ have suggested that a trial sequence where an incorrect trial is followed by a correct one is more instructive than a sequence of correct trials. Consequently, we examined the relationship between previous and current trials in terms of uncertainty. We investigated neural uncertainty in relation to the type of the previous trial’s response. Investigation into whether previous responses influenced present neural uncertainty, as confidence levels ascended during learning, also disclosed no substantial differences, in both naive (Fig. 5a, ANOVA, *P* = 0.57) and expert learners (Fig. 5c, ANOVA, *P* = 0.16).

To delve further into the analysis, we calculated the neural uncertainty based on the pairings of previous and current trial response types. In this spider plot, the lines represent the responses of the current trial and the axes correspond to the responses of the previous trial. Contrary to our expectations in the naive phase, where instructional pairings were presumed to produce marked differences in neural uncertainty, only a few pairs showed significant differences (Fig. 5b). The neural uncertainty was significantly greater when a miss followed a RO (*P* = 0.006) and significantly lower following an UR (*P* = 0.006). This suggests that, in fS1, there is a maintenance of high overall neural uncertainty during the early stages of learning, potentially driving the learning process without substantially differentiating the uncertainty of individual experiences.

Next, we examined the expert mice. We observed significant differences in neural uncertainty in current trials when the preceding trials were classified as miss, RO or UR (Fig. 5d). It seems plausible that RO may increase uncertainty in incorrect trials and decrease it in correct ones, whereas UR could potentially reduce uncertainty in incorrect trials and increase it in correct ones. This observation is noteworthy as it implies that RO and UR are set to serve contrasting roles (Fig. 5e). Therefore, we confirmed that experiencing a result significantly deviating from previous predictions leads to a change in the current uncertainty. Moreover, it seems reasonable to speculate that, after the learning period, there might be a differentiated assessment of the uncertainty related to individual experiences. Therefore, it can also be considered that uncertainty originates from what is known.

## Discussion

In an uncertain world, we use uncertainty to guide information searches^1^, evaluate data^2^ and make decisions. This process is crucial in the brain’s primary sensory cortex, which handles sensory information. Although traditionally challenging to quantify uncertainty, recent deep learning advancements have enabled new methods for its measurement^10^. Our study uses a transformer model without positional encoding to introduce a groundbreaking approach for decoding neural representations. This method generalizes across different neural activity patterns and sessions without positional cues, overcoming traditional limitations that required analyzing the same neurons over multiple days. By adopting these advanced techniques, we provide a more effective and practical framework for neural decoding that moves beyond the constraints of previous models reliant on cell tracking.

Uncertainty can arise from the complexity, variability and irregularity of neural activity itself, and if our model accurately reflects this, we can consider that our model reasonably reflects neural uncertainty. Uncertainty may also emerge from data acquisition or the data itself, such as from the low resolution of the device or noise from the device itself or the calcium indicator. As this occurs across all data, it can be relatively resolved through comparative analysis like in this Article. The uncertainty that arises from the model is due to insufficient learning of the data. However, the robust performance of our model allows us to largely mitigate this issue. Therefore, we believe that there is sufficient evidence to suggest that our results reflect neural uncertainty.

In this study, Slc17a7;Ai93;CaMKIIa-tTA transgenic mice were used. Contrary to findings reported in ref. ^20^, our mice did not exhibit neurons that were accurately activated by low-frequency oscillatory stimuli. Moving forward, it is considered necessary to undertake experiments within lines where GCaMP is expressed in all neurons, allowing the precise identification of neurons that accurately respond to low-frequency vibrational stimuli, thus overcoming the previously mentioned limitations.

We have pioneered an approach that “quantifies uncertainty within neural data”, providing a potentially invaluable tool for conducting more profound analyses of neural activity in states of uncertainty. Our findings demonstrate that the results of neural UQ across various biological situations (including learning processes, stimulus ambiguity, interruptions in learning and appropriateness of decision-making) are consistent with the biological perspectives of existing research, thereby validating this methodology as an appropriate tool for biological interpretation. Moreover, this study discovered that the neural representation of uncertainty dynamically changes between naive and expert states.

Our study has explored various aspects of uncertainty. We were able to observe not only stimulus-dependent uncertainty, as shown in Supplementary Fig. 7, but also decision-dependent uncertainty, as depicted in Fig. 3. The similarities between these two forms of uncertainty suggest a correlation between stimulus-dependent and decision-dependent uncertainties. This indicates that uncertainty in S1 arises not merely from a single factor but from an integration of multiple complex factors. Visual cortex neural activity can predict perceptual decisions—a relationship known as choice probability—that can be influenced and altered by top-down signals from areas such as the lateral intraparietal area or the frontal eye field^34^. It has also been suggested that uncertainty may be encoded in population codes^21^. Thus, the activity of the sensory circuit is altered by the integration circuit, leading to changes in sensory cortex perceptual or decision uncertainty through changes in population encoding, similar to the learning-dependent uncertainty discussed in this Article. This extends previous research showing that S1 encodes not only stimuli but also rewards and decisions, advancing our understanding that uncertainty in S1 dynamically and integratively changes, influenced by a combination of complex factors, beyond simple stimulus-dependent uncertainty.

We believe that our research, through the analysis of mechanisms using UQ from neural data, can serve as a catalyst for enhancing our understanding in various disorders where uncertainty plays a significant role. For example, our methodology can be applied to research on neurotransmitters such as dopamine^35^ and norepinephrine^36^, which are known to be related to uncertainty. Furthermore, our framework can be applied to diseases known to be associated with uncertainty, such as schizophrenia^37^ and autism spectrum disorder^38,39^. This study investigates new aspects of neural uncertainty representation and applies advanced deep learning techniques to analyze neural data. In addition, in the Supplementary Information, we demonstrate that the methodology presented in this Article aligns more closely with the biological context and exhibits superior performance compared with existing methods. Overall, our work not only explores new territories in the neural representation of uncertainty but also introduces innovative methods in deep learning, paving the way for future interdisciplinary research in these dynamic fields.

## Supplementary information

Supplementary Information

## Data Availability

The data that support the findings of this study are available from the corresponding authors upon reasonable request.

## Code availability

The code used in this Article is available via GitHub at https://github.com/toparkshin/s1uq_public.
