# Supplementary material for: Quantitative dynamics of neural uncertainty in sensory processing and decision-making during discriminative learning
Source: Exp Mol Med. 2025 May 7;57(5):1078–88. doi: 10.1038/s12276-025-01456-7 (PMC12130215; doi:10.1038/s12276-025-01456-7)
Supplement: Supplementary file 1 — Supplementary Information [file 12276_2025_1456_MOESM1_ESM.pdf]

This Supplementary Material has been prepared to provide additional insights into the main text. The contents are as follows:

1. **Pairwise p-value Matrix**
2. **Comparison of Psychometric Curves across Multiple Conditions**
3. **Decoding Accuracy and Behavioral Correlations**
4. **The Lick Proportion of Mice for Each Stimulus across Days**
5. **The Relationship between Response Time and Neural Uncertainty**
6. **Changes in Uncertainty for 'Hit' across Days**
7. **Uncertainty by Lick Status across Vibration Frequencies**
8. **Stimulus Uncertainty**
9. **Inference Values in Response Decoding**
10. **Comparison between Traditional Methods and the Neuron Transformer**
11. **Detailed information on the Neuron Transformer**

1. Pairwise p-value Matrix

Here is the pairwise p-value matrix for the statistics in the text.

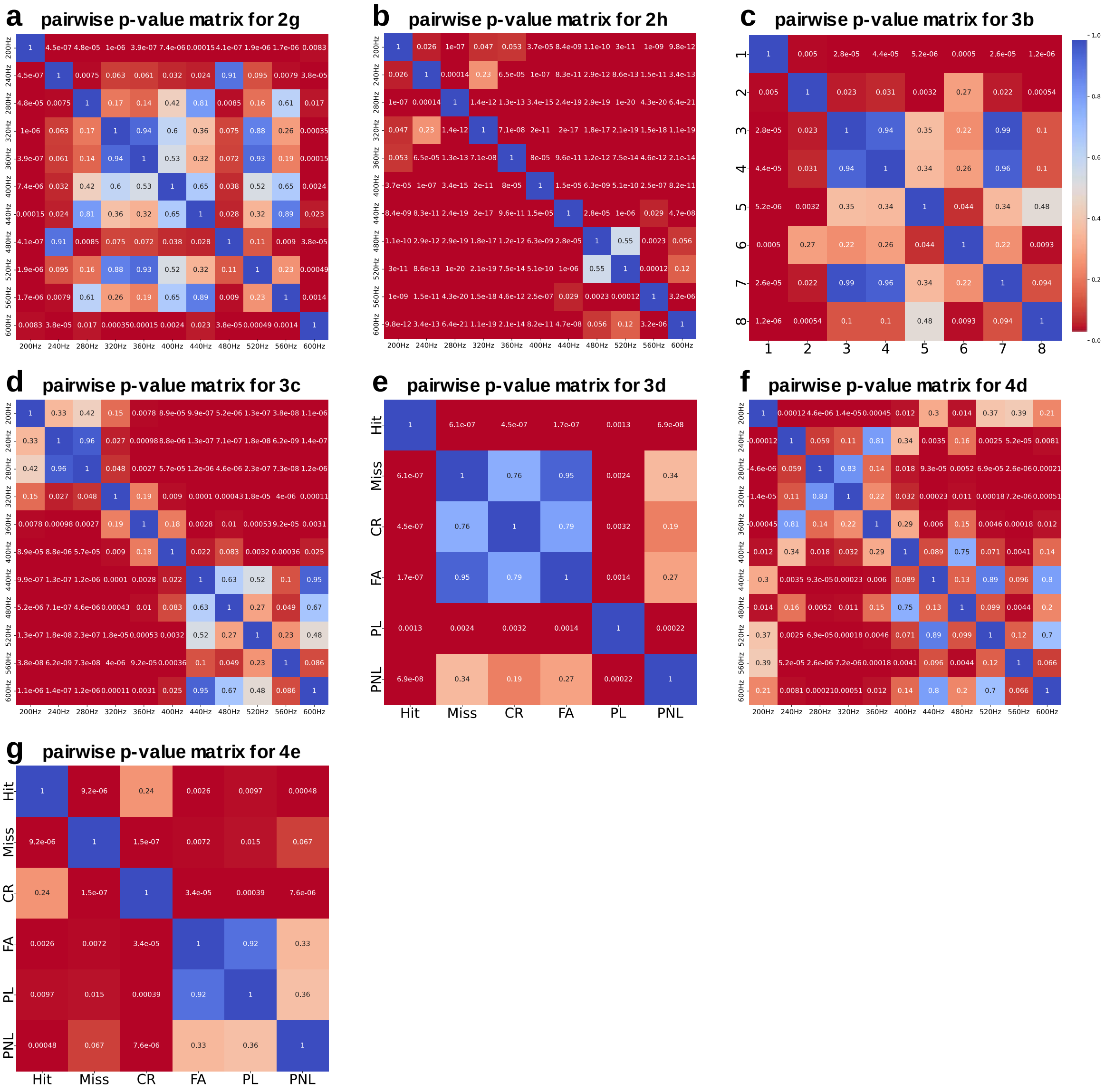

Supplementary Fig. 1. Pairwise p-value matrix. For the pairwise p-value matrix corresponding to each graph in the text.

2. Comparison of Psychometric Curves across Multiple Conditions

To investigate potential differences in the psychometric curves within a single day, we compared the Imaging Session with the subsequent Additional Session (Supplementary Fig. 2a). While the psychometric curve appears to show a slight rightward shift, no statistically significant differences were observed between the two conditions (paired t-test, mean p-value: 0.988). It is possible that the absence of significance is due to the small sample size (n = 16).

Additionally, we compared the psychometric curves of naive and expert mice (Supplementary Fig. 2b). In this comparison, we observed a significant rightward shift in the psychometric curve of expert mice relative to naive mice (paired t-test, mean p-value: 0.0126). This result suggests that expert mice perceive the cost of punishment as more substantial than the absence of cost associated with licking during probe trials.

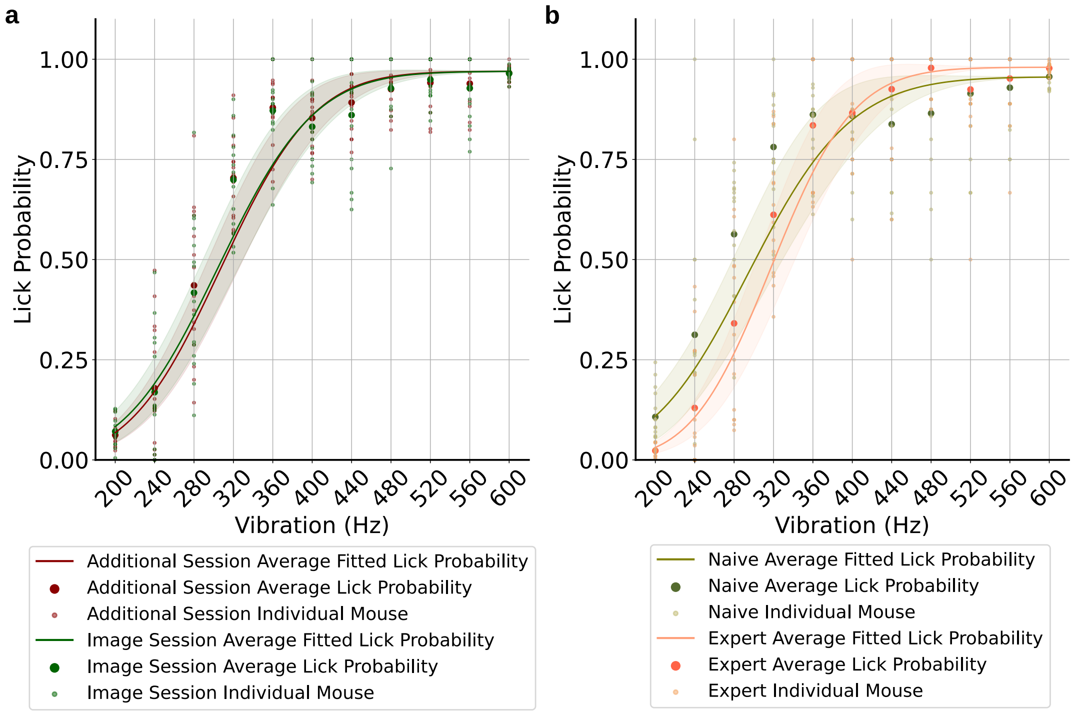

Supplementary Fig. 2. Comparison of psychometric curves across multiple conditions. a. No significant difference was detected in psychometric curves between the 'additional session' and 'Image session' conditions (paired t-test, mean p-value: 0.988). b. Notable differences in psychometric curves emerged when comparing naive to expert mice (paired t-test, mean p-value: 0.0126)

3. Decoding Accuracy and Behavioral Correlations

To investigate whether stimulus decoding is predictive of mouse behavior, we analyzed the relationship between decoding accuracy and behavioral responses, specifically focusing on whether incorrect stimulus decoding corresponds to false alarm or miss trials.

First, we classified trials based on decoding accuracy. Trials were labeled as "incorrect decoding" when the ground truth label did not match the model's prediction. For these incorrectly decoded trials, we examined the behavioral response to determine the frequency of false alarms and misses. Among all trials analyzed, misses accounted for 2.37% and false alarms for 6.20%. Within the correctly decoded trials, misses were 1.89% and false alarms were 0.08%. Therefore, it can be observed that there were a relatively higher number of misses and false alarms among the incorrectly decoded trials. Among the incorrectly decoded trials, the proportion of false alarms was 6.5%, and the proportion of misses was 4.1%. These results suggest that incorrect stimulus decoding is somewhat associated with false alarm or miss behaviors. However, as these proportions also include responses during probe trials, additional analysis was conducted to refine this relationship.

We next analyzed whether the probability of incorrect stimulus decoding was higher during miss or false alarm trials. This analysis specifically evaluated decoding accuracy within miss and false alarm cases to assess whether decoding errors were more likely during these behavioral events. We found that 55% of miss trials and 52.3% of false alarm trials involved incorrect stimulus decoding. Therefore, it was observed that there were considerable instances of incorrect behavior when the stimulus was misjudged. These findings indicate that incorrect decoding is predictive of miss and false alarm behaviors.

4. The Lick Proportion of Mice for Each Stimulus across Days

The following graph (Supplementary Fig. 3) illustrates the lick proportion for each mouse across training days for different stimuli: nogo, toss-up, go-like and go. For the go and go-like stimuli, the lick proportion increases rapidly over the first two days and then remains at a high level. In contrast, for the nogo stimulus, there is a steep decline in lick proportion. Meanwhile, for the toss-up stimulus, the lick proportion decreases to some extent and then stabilizes.

a

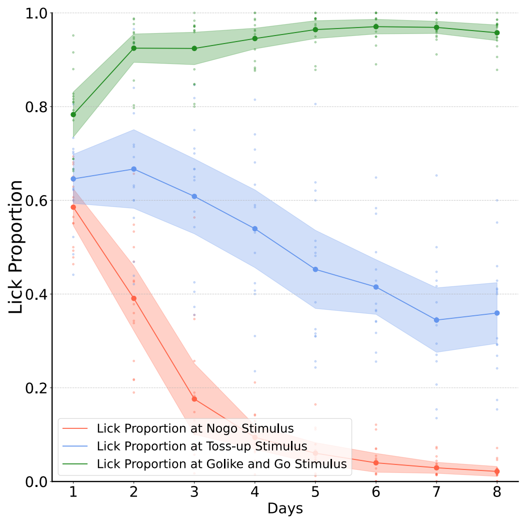

Supplementary Fig. 3. The lick proportion of mice for each stimulus across days

5. The Relationship between Response Time and Neural Uncertainty

Previous literature pointing to a correlation between response time (or decision time) and uncertainty[1,2] led us to an investigation into the connection between response time and uncertainty. Linear regression was used to assess this relationship across the data points. Subsequent calculations of the R^2 value and p-value for each mouse on each date indicated a lack of correlation (Supplementary Fig. 4). The trend observed in Fig 1g, with response times quickly reducing at the onset and forming a plateau, combined with the fact that prior studies differentiate based on intensity, not frequency, could potentially explain these findings.

a

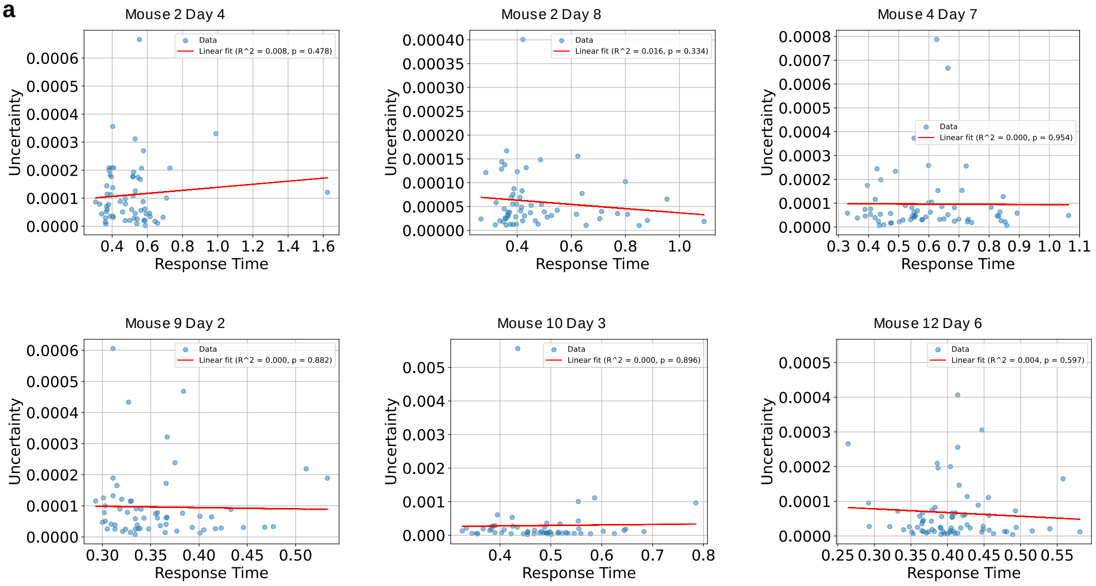

Supplementary Fig. 4. a. An illustration of the relationship between response time and uncertainty, where no significant difference was noted, contrary to expectations.

6. Changes in Uncertainty for 'Hit' across Days

Low uncertainty on hit trials could explain why uncertainty decreases over training days (Fig. 3b), and why it is highest near the perceptual threshold (Fig. 3c). To demonstrate that training days have an effect independent of hit rate, we produced the following graphs and confirmed this observation (Supplementary Fig. 5).

a

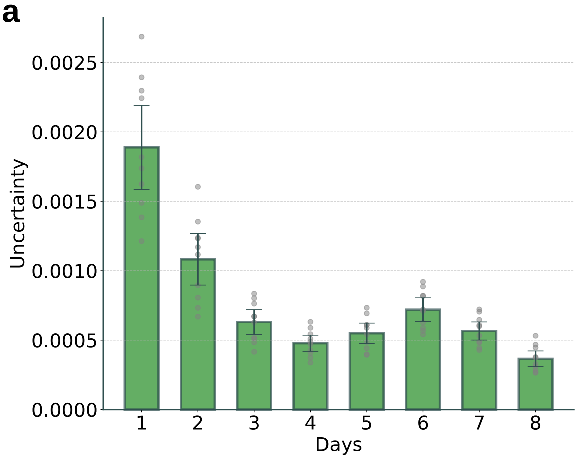

Supplementary Fig. 5. Changes in Uncertainty for 'Hit' across Days

7. Uncertainty by Lick Status across Vibration Frequencies

We analyzed the uncertainty, distinguishing between 'lick' and 'no lick' for each stimulus frequency. We anticipated higher uncertainty when there was a 'lick' in the toss-up stimulus and a 'no lick' in the go-like stimulus. While the results showed heightened uncertainty for 'lick' in the nogo stimulus, surprisingly, the toss-up stimulus revealed further increased uncertainty for 'no lick' (Supplementary Fig. 6a). In naive mice, the uncertainty associated with licking behavior, based on stimulus frequency, showed a smaller difference between 'lick' and 'no lick' for the go-like stimulus compared to the total phase. Furthermore, higher uncertainty was observed in the no lick cases for both go and nogo stimuli (Supplementary Fig. 6b). In expert mice, we observed higher uncertainty during licks in response to nogo and toss-up stimuli (Supplementary Fig. 6c). Thus, in the expert mice, higher uncertainty was observed for stimuli closer to nogo on the psychometric curve when licking occurred, and for stimuli closer to go when no licking occurred, which aligns with our expectations.

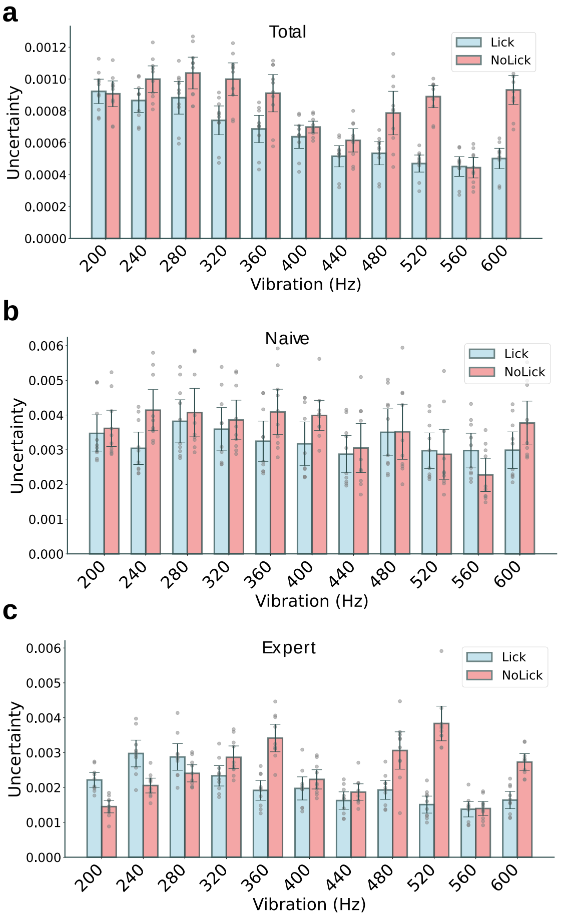

Supplementary Fig. 6. Uncertainty by lick status across vibration frequencies: total in **(a)**, naive in **(b)**, expert in **(c)**.

8. Stimulus Neural Uncertainty Results

Stimulus neural uncertainty was calculated similarly to response neural uncertainty, with labels set to 0 for 200 Hz and 1 for 600 Hz. Upon examining the stimulus neural uncertainty, we observed a decline in uncertainty up to day 5, followed by an increase on day 6, and then a subsequent decrease (Supplementary Fig. 7b; ANOVA: F-statistics: 9.115, p-value: 5.16×10^{-8}). The observed uptick in uncertainty on Day 6 is believed to stem from the pause in behavioral experiments over the weekend. Given that neither the mice's behavioral performance nor the deep learning model's accuracy showed a decline on Day 6.

Next, we examined the stimulus uncertainty for each frequency. This represents the uncertainty when determining how close each stimulus is to either 200 Hz or 600 Hz. We observed that the uncertainty is elevated at the toss-up frequency (Supplementary Fig. 7c; ANOVA: F-statistics: 42.436, p-value: 2.14×10^{-31}).

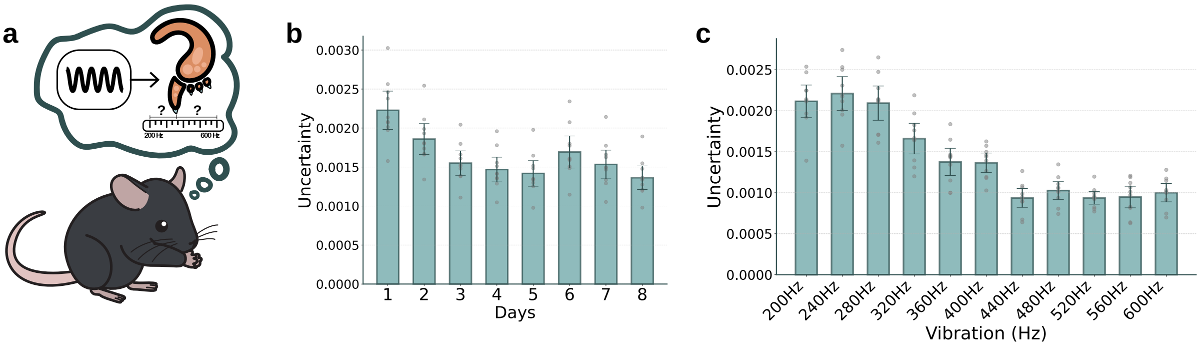

Supplementary Fig. 7. a. An illustration of the concepts of uncertainty in stimulus uncertainty. The illustrative representation of the uncertainty in fS1, evaluating how mice perceive vibrations as being closer to 600Hz or 200Hz. This concept is portrayed using a paw-shaped question mark, indicating their respective positions on the psychometric scale. **b.** Uncertainty of stimuli across dates. A consistent decline in uncertainty, with an uptick on the sixth day attributed to a pause in experimentation over the weekend. **c.** Uncertainty of stimuli by vibration frequency.

9. Inference Values in Response Decoding

In response decoding, we visualized the distribution of inference values using graphs (Supplementary Fig. 8a, b). Importantly, this figure does not reflect variance across individual trials and thus does not indicate uncertainty; it simply shows the distribution of inferences. Notably, at the toss-up frequency, unlike other frequencies, we observed values at both extremes in the violin plot, as shown in panel a. Additionally, in panel b's box plot, the upper and lower 75th percentiles were more widely distributed compared to other frequencies. Further analysis by date and frequency (Supplementary Fig. 8c) revealed that the toss-up frequency consistently exhibited a wide distribution, whereas other frequencies showed a narrowing distribution over time. This suggests that mouse behavior at the toss-up frequency has high variability, and as learning progresses, the behavioral variability remains high at the toss-up frequency but decreases at other frequencies, indicating that our model effectively captures these trends.

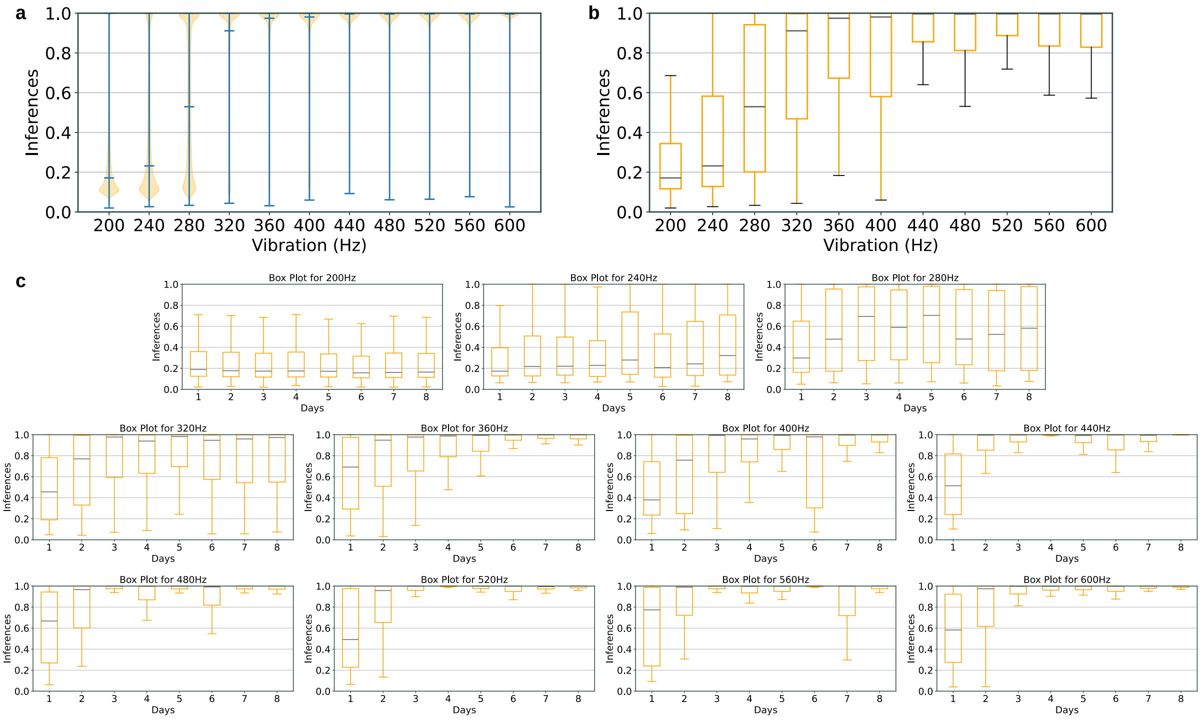

Supplementary Fig. 8. a. Violin plot of inference values. **b.** Box plot of inference values. **c.** Box plots showing the distribution of inference values by date for each frequency.

10. Comparison between Traditional Methods and the Neuron Transformer

10-1. Trial-by-trial Variability

For trial-by-trial variability, due to the difficulty of calculating across different days' datasets, we employed the following method. We utilized the z-scored neural activity from frames 29 to 37 for each trial and calculated the trial-by-trial variability for these identical frames across trials, averaging these values per trial. The trial-by-trial variability for each day was computed for each mouse and each day (Supplementary Fig. 9a). For the trial-by-trial variability related to responses, we calculated the variability for each response type per day for each mouse (Supplementary Fig. 9b) and then averaged these across responses for each mouse. When conducting ANOVA, no significant differences were found either by day (ANOVA; F-statistic: 1.17, p-value: 0.326) or by response type (ANOVA; F-statistic: 0.13, p-value: 0.985). Therefore, it is determined that directly measuring uncertainty through trial-by-trial variability is not appropriate.

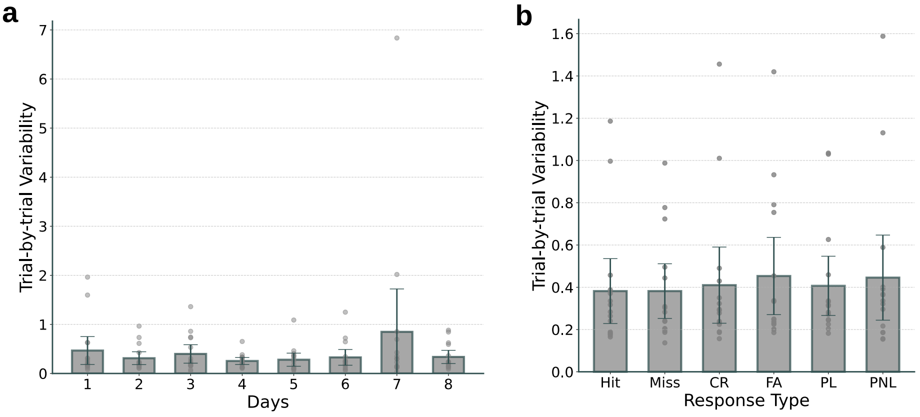

Supplementary Fig. 9. a. Trial-by-trial variability across days. **b.** Trial-by-trial variability across response types.

10-2. Fano Factor

Similarly, we calculated the Fano factor (variance/mean) for the z-scored neural activity across 9 frames. For each mouse, we computed the Fano factor based on response and day, and checked for significant differences in the Fano factor across these categories. We found no significant differences between Hits and Misses (Mann-Whitney U test; p-value: 0.2628), but a significant difference was observed between Correct Rejects and False Alarms (Mann-Whitney U test; p-value: 0.0310). Additionally, we checked for significant differences in the Fano factor across days, but no significant differences were found (ANOVA; F-statistic: 0.84, p-value: 0.56). This indicates that Fano factor may not be an adequate predictor in this context.

10-3. Population-level Variance Metrics

We calculated population-level variance metrics to infer uncertainty, utilizing values from frames 29 to 37 of the z-scored neural activity. For each trial within the chosen frames, the variance of z-scored neural activity was calculated for each frame individually across all neurons. This step quantifies the spread of the activity values, reflecting the degree of synchronicity or variability in the neural ensemble's response. The variances obtained for each of the 9 frames (from 29 to 37) were then averaged to yield a single variance value per trial. To facilitate analysis across different conditions and sessions, these average variance values were grouped and further averaged according to the mouse's specific behavioral response and the experimental session day. The calculation of population-level variance serves as a robust indicator of the neural ensemble's coherence and variability in response to stimuli. Higher variance suggests less synchrony among neurons, potentially indicating a dispersed or varied response to the stimulus. Conversely, lower variance suggests a more uniform response across the neural population.

We observed no significant differences across days (Supplementary Fig. 10a, ANOVA; F-statistic: 1.25, p-value: 0.282), but significant differences were found between response types (ANOVA; F-statistic: 19.76, p-value: 5.76×10^{-13}). For Hit and Miss responses, Hit showed lower values which align with the biological context, while for CR and FA, CR values were higher, making them challenging to apply in the biological context (Supplementary Fig. 10b, c). This supports our assessment that simple activity variance is insufficient for evaluating uncertainty.

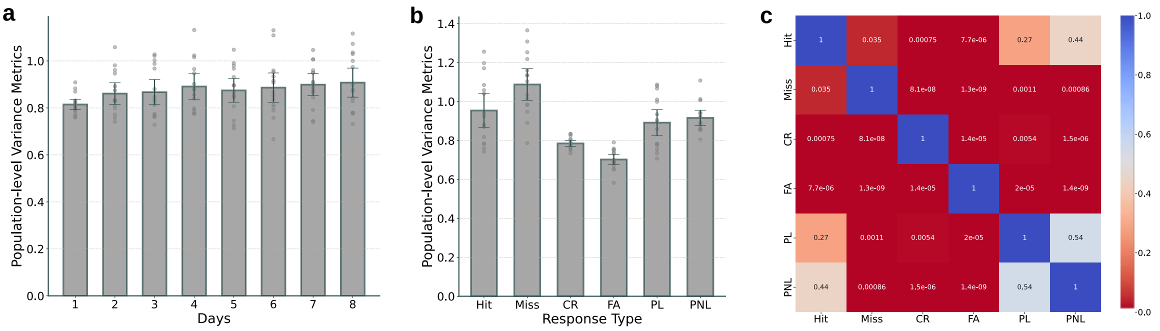

Supplementary Fig. 10. a. Population-level variance metrics across days. **b.** Population-level variance metrics across response types. **c.** P-value matrix for b.

10-4. Bayesian Logistic Regression

We employ Bayesian logistic regression, traditionally used for analyzing neural signals and estimating uncertainty[3,4], to predict binary outcomes (lick/nolick) from z-scored neural activity

data. Given neural activity data from multiple neurons over 29~37 frames per trial, our goal is to model the probability of a lick response based on these inputs.

Mathematical Formulation

Let yi represent the binary outcome for trial i, where yi = 1 indicates a lick event, and yi = 0 indicates no lick. The predictor variables xi encompass the z-scored neural activities from all recorded neurons across the 9 frames for trial i. The probability of a lick event given the neural activities is modeled using a logistic function:

$$\Pr(yi = 1|xi,\beta) = \frac{1}{1+\exp(-xi^T\beta)}$$

where β represents the vector of regression coefficients.

Prior Distribution

In the absence of prior domain-specific knowledge, we assign noninformative priors to the regression coefficients:

$$\beta j \sim \text{Normal}(0,\sigma^2)$$

where σ^2 is a hyperparameter representing the variance of the prior distribution, typically set to a large value to reflect our uncertainty about the coefficients.

Posterior Distribution

Bayesian inference is performed to update our beliefs about the coefficients β based on the observed data. The posterior distribution is proportional to the product of the likelihood and the prior:

$$p(\beta|y,X) \propto p(y|X,\beta)\ p(\beta)$$

where y and X denote the vector of outcomes and the matrix of predictors for all trials, respectively.

Computational Methods

We implemented the No-U-Turn Sampler (NUTS) for our Bayesian logistic regression model. Four independent MCMC chains were run to enhance the robustness and reliability of our parameter estimates. Each chain was initialized at different starting points and was run for 5,000 iterations. The first 1,000 iterations of each chain were discarded as burn-in to allow the chains to converge towards the stationary distribution.

To reduce autocorrelation and manage memory usage, we applied a thinning interval of 5, meaning that every tenth sample was retained for analysis. After thinning, we obtained a total of 800 effective samples per chain, resulting in 3,200 samples across all chains for posterior inference. Convergence was assessed using the Gelman-Rubin diagnostic statistic, with all values below 1.1, indicating satisfactory convergence for all model parameters. This calculation was performed separately for each mouse on each day.

Model Checking and Validation

Model validation is conducted via posterior predictive checks. We generate new data based on the posterior distribution of β and compare these predictions to the actual outcomes to assess model fit and predictive accuracy.

Uncertainty Quantification

Posterior Width (PW): We calculate the width of the 95% credible intervals for the regression coefficients to evaluate the uncertainty in our parameter estimates.

Results

The average accuracy of the model was 0.699, which was lower compared to 0.765 for the Neuron Transformer. No significant differences were observed in posterior width across days (Supplementary Fig. 11a, ANOVA; F-statistic: 0.509, p-value: 0.826). However, significant differences were found in posterior width according to response type (Supplementary Fig. 11b, c, ANOVA; F-statistic: 3.88, p-value: 0.003), with Hit showing significantly lower

posterior widths compared to other responses. This mirrored the results of our model. Further analysis on expert mice showed similar results, with lower posterior widths for Hit and CR (Supplementary Fig. 11d, e, ANOVA; F-statistic: 34.478, p-value: 5.48×10^{-19}), confirming that the Bayesian model can infer uncertainty appropriately within the biological context for data structured the same way on the same dates. However, it was not suitable for examining the overall learning process.

Through comparisons with various existing analytical methods, we confirmed that the Neuron Transformer is a more suitable tool for analyzing uncertainty and demonstrates better performance. This is significant as it overcomes the various limitations of traditional methods, as mentioned in the manuscript.

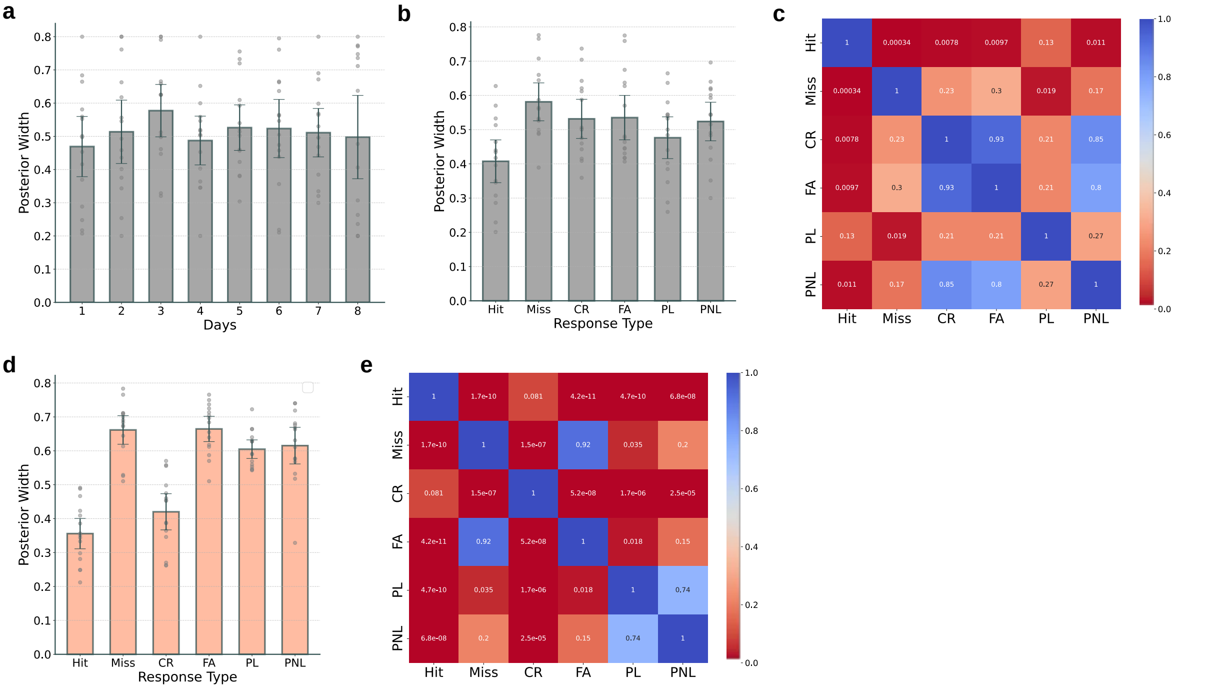

Supplementary Fig. 11. a. Posterior width across days. **b.** Posterior width across response types. **c.** P-value matrix for b. **d.** Posterior width of expert mice regarding response types. **e.** P-value matrix for d.

10-5. Single-neuron Ideal Observer

To evaluate the performance of the Neuron Transformer through comparison with univariate decoding, we compared our model with the Single-neuron Ideal Observer approach, which was previously used to analyze two-photon calcium imaging data from the somatosensory cortex in a previous study[5]. Subsequent analyses were also conducted using frames 29 to 37 (9 frames).

Single-neuron Ideal Observer ROC Analysis

Since the number and identity of neurons remained the same within each mouse on each day, we performed a single-neuron ideal observer analysis. We used ROC analysis to calculate detect probability (choice probability) and stimulus probability. A decision variable (DV) was assigned based on neural responses, and the area under the ROC curve (AUC) was used to evaluate trial categorization. Detect probability corresponds to the AUC for discriminating the mouse's choice (lick vs. no-lick), while stimulus probability corresponds to the AUC for distinguishing between 200 Hz and 600 Hz vibration stimuli. The neuron with the highest AUC was selected as the ROC curve for the corresponding mouse on that day and was later used for comparison with the Neuron Transformer.

Neuron Transformer ROC Analysis

We trained the Neuron Transformer excluding a specific mouse's specific day and then performed inference on the dataset created for that particular mouse on that specific day to obtain the ROC curve. At this stage, we performed the analysis to distinguish between lick and no-lick, as well as 600 Hz and 200 Hz, following the same approach as the previous response decoding and stimulus decoding methods. This allowed us to generate an ROC curve for each mouse on each day, similar to the single-neuron analysis.

Results

To compare these two, we averaged the ROC curves for each mouse on each date from each decoding, and compared these with a display of the 95% confidence interval (Supplementary Fig. 12). In response decoding, the single-neuron ideal observer had an average of 0.788 and a variance of 0.0114, while the Neuron Transformer had an average of 0.854 and a variance of 0.0139. In stimulus decoding, the single-neuron ideal observer had an average of 0.868 and a variance of 0.00974, whereas the Neuron Transformer had an average of 0.933 and a variance of 0.00595. In both response and stimulus decoding, the AUCs were significantly higher for the Neuron Transformer (paired t-test; T-Statistic: -8.50, p-value: 6.51×10^{-14} for response and T-Statistic: -9.07, p-value: 2.99×10^{-15} for stimulus). These results confirm that the Neuron Transformer can deliver sufficient performance even with smaller datasets compared to traditional methods.

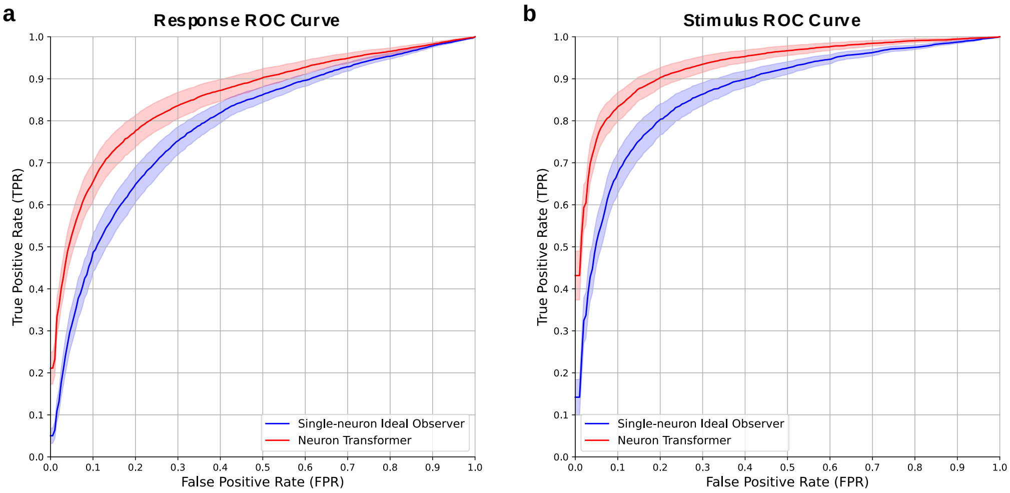

Supplementary Fig. 12. a. The ROC curves of the Single-neuron ideal observer and Neuron Transformer in response decoding. **b.** The ROC curves of the Single-neuron ideal observer and Neuron Transformer in stimulus decoding.

10-6 Random Forest

For each mouse and each day, we trained a random forest classifier to predict lick behavior using 9-frame z-scored neural activity. The test size was set to 0.3, and the random state was varied across 10 different configurations. The model used 100 estimators with a minimum leaf size of 1. The average accuracy was 0.722, which was lower than the 0.765 accuracy of the Neuron Transformer (t-test; T-Statistic: 9.739, p-value: 1.61×10^{-8}).

11. Neuron Transformer

This Supplementary Material covers the structure of the Neuron Transformer, data handling, training, and other detailed aspects (Supplementary Fig. 13).

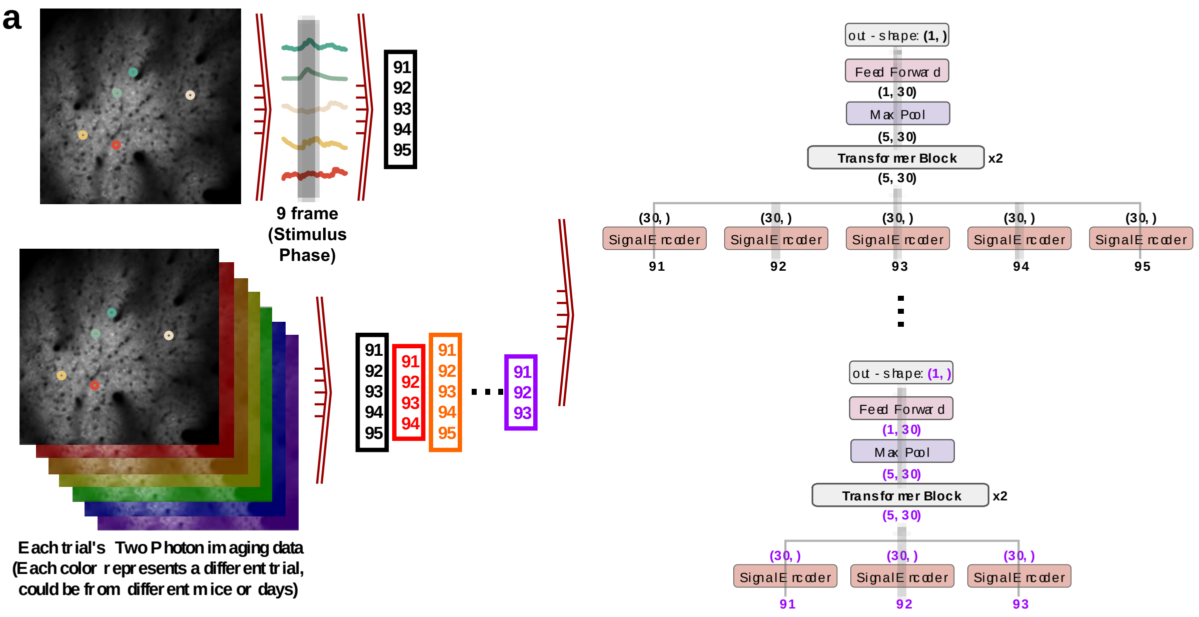

Supplementary Fig. 13. Data Processing Flow Chart for Uncertainty Quantification Using the Neuron Transformer. The subscripts 1, 2, 3, 4 accompanying numbers 9_1, 9_2, 9_3, 9_4 represent individual neuron identifiers captured within the same trial, with different colors indicating distinct trials that include varying dates and mice. And the number 9 represents the data points across 9 frames. This data is input into a transformer structure as depicted on the right side of the figure, accommodating data from each neuron of every trial. The architectural flexibility of the transformer, illustrated on the right, allows for handling inputs of varying sizes, as demonstrated by trials like the black one with five neurons and the purple one with three neurons.

Data Acquisition and Preprocessing

Neural activity data was obtained from the fS1 of mice performing a go/nogo task, using two-photon calcium imaging with GCaMP6 indicators. We employed the CaImAn toolkit for the preprocessing of 2p images, which included steps such as neutrophil subtraction and movement correction to accurately derive neural activities. Subsequently, we calculated the z-scored neural activity from each trial to proceed with the following analyses.

Each trial's imaging data was used only during the stimulus presentation phase, specifically from frames 29 to 37 post-stimulus onset, corresponding to 0.233 seconds. This period precedes the established minimum response time of 0.273 seconds (determined post-training from Day 4 onwards), ensuring that the captured neural activity was independent of motor responses. Each of these frames was min-max scaled and segmented into 9-frame portions to form structured datasets. Labels were assigned based on the stimulus or response type during these trials; for stimuli, 200 Hz was labeled as 0 and 600 Hz as 1, while for responses, 'no lick' was labeled as 0 and 'lick' as 1.

Data Structuring for Neural Network Training

For the purposes of training our neural network models, we structured the data as follows:

- *Data*: A list containing 1000 trial elements, where each element is an ndarray of shape (200, 120), representing the fluorescence activity from 200 neurons over 120 frames on each trial (4 seconds at 30fps). 120 frames encompass the entire trial period, and the publicly available code includes the process of slicing out the 9 data points needed for analysis.
- *Stimulus*: An ndarray of shape (1000,) categorizing the type of stimulus (e.g., Go, Nogo, Probe) as integers.
- *Response*: An ndarray of shape (1000,) detailing the mouse's behavioral response (e.g., Hit, Miss, Correct Rejection, False Alarm) as integers.
- *Frequency*: An ndarray of shape (1000,) listing the vibration stimulus frequencies.
- *Day Info*: An ndarray of shape (1000,) indicating the day of the behavioral experiment.
- *Trial Info*: An ndarray of shape (1000,) marking the trial number for each session.
- *Mouse Info*: An ndarray of shape (1000,) denoting each mouse as integers.
- *Label*: An ndarray of shape (1000,) where labels are set as 0 or 1 based on the stimulus or response type.

The primary data components for learning are 'data' and 'label', with the rest aiding in organizing and analyzing results across different conditions and trials.

Dataset Partitioning

For effective model validation and testing, the data was partitioned into train, validation, and test sets. The proportions for the train, validation, and test sets are 76%, 19%, and 5%, respectively.

Neural Network Architecture

1. Signal Encoder

The architecture initiates with a Signal Encoder that employs a feed-forward neural network to transform raw input data into a reduced dimensional latent space. This transformation is pivotal for condensing the complex neural data into a manageable form for subsequent layers.

Configuration: The encoder consists of a linear layer that maps input data dimensions to a latent space, typically ranging from 9 to 30 dimensions based on experimental requirements.

Purpose: This step is essential for reducing the complexity of the input data, facilitating more efficient processing in the deeper layers of the model.

2. Transformer Blocks

At the core of our architecture are Transformer blocks, designed to handle sequences of data by capturing temporal dynamics and dependencies within the neural activities.

Self-Attention Mechanism: Each block features a self-attention mechanism that assigns adaptively weighted importance to different segments of the input sequence. This mechanism allows the model to focus on relevant temporal features that are crucial for prediction.

Layer Composition: The structure of each Transformer block includes:

A multi-head self-attention layer that processes the data in parallel, capturing various aspects of the input information.

Normalization and Non-linearity: Post self-attention and feed-forward processing, each component's output is normalized using layer normalization to stabilize training. The Gaussian Error Linear Unit (GELU) activation function is applied to introduce necessary non-linear processing capabilities.

3. Post-Transformer Layers

After the sequence has been processed through the transformer blocks, further layers refine and prepare the data for predictions:

Max Pooling: This operation condenses the transformer block's output along the sequence dimension (number of neurons in the input data) . It is particularly effective for handling variable-length data by reducing the output to a consistent format, typically to the shape of (Batch Size, Latent Dimension). This step is crucial for capturing the most significant features from the varied sequence lengths. The order of neurons is not important because the positional encoder has been omitted.

Feed Forward: Following max pooling, the data is passed through another 2-layer feed-forward network. This network processes the pooled output to refine the features for the final prediction layer.

Training Process for Decoding

The training regimen was structured as follows:

Batch Sizes: The model was trained with a batch size of 64 for training, 16 for validation, and 16 for testing, optimizing the balance between memory constraints and model performance.

Dropout Configurations: To mitigate overfitting, node dropout was implemented at different stages of the model (See Fig 2a):

Embedding Dropout: 0.1 (applied to Signal Encoder)
Residual Dropout: 0.1 (applied in Transformer Block to Key and Value)
Attention Dropout: 0.1 (applied at the final stage of Transformer Block)

Learning Rate and Iterations: The training was conducted with a learning rate of 0.0001 across 20,000 iterations. We employed a strategy to keep the model version exhibiting the minimal cross-entropy loss observed during the validation phase for subsequent testing.

Initialization and Weight Adjustments: We meticulously initialized the network weights and adjusted them as necessary during training to optimize performance and convergence. We used Adam as the optimizer.

This training strategy ensures that the model learns effectively from the training data, maximizing its ability to generalize from the training to the testing scenarios. It also allows the model to capture subtle nuances in the data which are essential for accurate uncertainty quantification.

Uncertainty Quantification

We implemented a k-fold cross-validation approach to estimate uncertainties, dividing the dataset into 20 folds for focused analyses on stimuli of 200 Hz and 600 Hz. Each fold excluded trials with premovement activities, using 80% of the remaining data for training and 20% for validation. Therefore, the proportions for the train, validation, and test sets are 76%, 19%, and 5%, respectively. This model setup was used to infer uncertainties in the test dataset, repeating the process 10 times with different random seeds for partitioning and data input to ensure comprehensive uncertainty analysis and robustness of the findings. During testing, dropout was enabled, and after conducting 1000 iterations on each dataset, uncertainty was quantified by calculating the variance of the inferences.

This structured and detailed preparation ensures that our neural network models are well-equipped to accurately quantify and interpret neural responses to varying stimuli and responses, isolating sensory processing from motor responses and providing a nuanced understanding of neural dynamics in decision-making tasks.

1. Madisen, L. *et al.* Transgenic Mice for Intersectional Targeting of Neural Sensors and Effectors with High Specificity and Performance. *Neuron* **85**, 942–958 (2015).
2. Heitz, R. P. The speed-accuracy tradeoff: history, physiology, methodology, and behavior. *Front. Neurosci.* **8**, 150 (2014).
3. Jegminat, J., Jastrzębowska, M. A., Pachai, M. V., Herzog, M. H. & Pfister, J.-P. Bayesian regression explains how human participants handle parameter uncertainty. *PLoS Comput. Biol.* **16**, e1007886 (2020).
4. Cao, X., Lee, K. & Huang, Q. Bayesian variable selection in logistic regression with application to whole-brain functional connectivity analysis for Parkinson's disease. *Stat. Methods Méd. Res.* **30**, 826–842 (2021).
5. Kwon, S. E., Yang, H., Minamisawa, G. & O'Connor, D. H. Sensory and decision-related activity propagate in a cortical feedback loop during touch perception. *Nat. Neurosci.* **19**, 1243–1249 (2016).
